# Evaluation of social protection for people affected by tuberculosis: development of an indicator matrix for Brazil

**DOI:** 10.1186/s12889-025-24689-7

**Published:** 2025-11-25

**Authors:** Melisane Regina Lima Ferreira, Paula Daniella de Abreu, Venisse Paschoalin Maurin, Rubia Laine de Paula Andrade-Gonçalves, Tiemi Arakawa, Jaqueline Garcia de Almeida Ballestero, Ricardo Alexandre Arcêncio, Inês Fronteira, Aline Aparecida Monroe

**Affiliations:** 1 University of São Paulo at Ribeirão Preto College of Nursing, Ribeirão Preto, São Paulo, Brazil; 2 General Coordination for Tuberculosis Surveillance, Endemic Mycoses, and Nontuberculous Mycobacteria, Department of HIV/AIDS, Viral Hepatitis and Sexually Transmitted Infections, Secretariat of Health and Environmental Surveillance (CGTM/DATHI/SVSA), Ministry of Health, Federal District, Brasília, Brazil; 3 National School of Public Health, Public Health Research Center, Comprehensive Health Research Center, NOVA University Lisbon, Lisbon, Portugal

**Keywords:** Tuberculosis, Public policy, Social welfare, Healthcare quality indicator, Human rights, Delphi technique

## Abstract

**Background:**

In the context of tuberculosis elimination, social protection seeks to mitigate multiple forms of vulnerability experienced by people affected by the disease, addressing both biological and social factors and directly targeting the social determinants of health. However, in Brazil, the absence of standardized conceptual tools and process indicators to assess the operationalization and implementation of intersectoral actions and services related to social protection for tuberculosis control highlights the need for an instrument capable of evaluating how the social protection network functions for people affected by tuberculosis.

**Methods:**

This methodological study was conducted in Brazil to develop and validate an instrument comprising indicators for measuring and assessing the implementation of social protection actions for people affected by tuberculosis. In the first stage, a literature review and document analysis were carried out, resulting in the development of a Logic Model to guide the construction of the Evaluative Matrix. The second stage involved content validation of the matrix using the Delphi method, with the aim of reaching satisfactory Content Validity Indices (CVI > 0.80) through expert consensus. Nine experts participated in the first round and seven in the second.

**Results:**

The Evaluative Matrix was validated with satisfactory CVI scores, demonstrating significant expert consensus. It offers a comprehensive mapping of the national landscape of social protection for people affected by tuberculosis. The instrument comprises 20 evaluative categories and 53 indicators, each with defined parameters and data sources, distributed across 11 subdimensions derived from four main dimensions of the Logic Model: (I) the right to health; (II) the right to social assistance; (III) the right to social security; and (IV) shared responsibilities.

**Conclusions:**

This study presents an innovative evaluation tool to support managers and decision-makers in designing and improving social protection strategies within health policies for people affected by tuberculosis, while accounting for diverse local contexts. It aims to accelerate progress toward tuberculosis elimination by addressing poverty and other social determinants, and by protecting the human rights of people with tuberculosis and their families.

**Supplementary Information:**

The online version contains supplementary material available at 10.1186/s12889-025-24689-7.

## Background

Globally, the context of social inequalities and health vulnerabilities surrounding the occurrence and outcomes of tuberculosis (TB) has been exacerbated by the sanitary, financial, and geopolitical crises triggered by the COVID-19 pandemic. This scenario has made the implementation of intersectoral policies that address the social determinants of the health–disease–outcome process an urgent priority, with impacts on the health of people affected by TB, on controlling transmissibility, and on reducing the economic burden generated by the disease [1, 2].

Recognized as a socially (re)produced disease, TB was estimated to have affected 10.6 million people and caused over 1.3 million deaths worldwide in 2022, particularly in less developed countries, where 95% of TB deaths occur [3]. In Brazil, during the same year, 78,057 new cases of TB and 5,162 TB-related deaths were reported, placing the country as the only one in the Americas region and 20^th^ globally among the 30 countries with the highest TB incidence rates, thus making it a priority for TB prevention and care initiatives worldwide [4].

Major international agreements have set ambitious targets for ending the global TB epidemic. Notably, the Sustainable Development Goals (SDGs), established by the United Nations (UN) in 2015, aim to end the TB epidemic by 2030. Similarly, the World Health Organization’s (WHO) End TB Strategy, launched in 2015, sets targets of a 90% reduction in TB incidence and a 95% reduction in TB mortality by 2035, along with the elimination of catastrophic costs associated with the disease [5]. These goals are underpinned by the strengthening of bold policies and support systems for people affected by TB and their families [3].

In Brazil, the National Plan for Ending TB as a Public Health Problem, developed in 2017 and revised in 2021 [6], aligns with WHO international agreements. More recently, in 2023, Brazil established the Interministerial Committee for the Elimination of Tuberculosis and Other Socially Determined Diseases (CIEDDS), aiming to accelerate efforts to meet operational targets for TB elimination, anticipating the deadline to 2030 [7].

Social protection, recognized as a human right, is defined as a set of policies and programs designed to safeguard lives and human rights, prevent poverty, and mitigate risks and vulnerabilities across the life course [8]. In Brazil, social protection is extensively regulated by the 1988 Federal Constitution and is an integral component of the social security system, which is structured around the pillars of health, social assistance, and social security policies [9].

This system is funded through a combination of employee and employer contributions, taxes, and specific public funds, and aims to reduce social inequalities, promote well-being, and combat social exclusion, despite historical challenges and setbacks [9]. Health is universally guaranteed through the Unified Health System (*Sistema Único de Saúde –* SUS), regulated by Law nº. 8.080/1990, which assigns to the State the responsibility for ensuring the conditions necessary for its full realization [10].

Social assistance was institutionalized through the Organic Law of Social Assistance (*Lei Orgânica da Assistência Social -* LOAS) and is implemented via the Unified Social Assistance System (*Sistema Único de Assistência Social – *SUAS), targeting individuals and families in situations of vulnerability, regardless of prior contributions [11, 12].

The Unified Registry (*Cadastro Único* - CadÚnico) is a strategic tool within the SUAS for identifying low-income families in Brazil. It enables their inclusion in a range of government-run social programs, such as the Bolsa Família Program (*Programa Bolsa Família* – BFP), the Continuous Cash Benefit (*Benefício de Prestação Continuada* – BPC), among others [11, 12].

Finally, the social security system is contributory and protects workers and their dependents against social risks such as disability, old age, maternity, unemployment, incarceration, and death. These benefits are managed by the National Institute of Social Security (*Instituto Nacional do Seguro Social* - INSS), which administers the General Social Security Regime, covering nearly 70% of Brazil’s working-age population in 2022 [9, 13].

In the context of TB control, social protection aims to mitigate various degrees of vulnerability experienced by people affected by the disease, encompassing both biological and social factors, and directly addressing the social determinants of health [14]. Moreover, a body of literature has shown that social protection measures and strategies improve nutritional status, quality of life, and treatment adherence, while also helping to reduce catastrophic costs associated with TB and promoting favorable treatment outcomes [15].

This highlights the role of social protection as a key link between health and social interventions. Its purpose goes beyond meeting programmatic goals - it aims to reduce poverty, promote equity and social justice, protect human rights, and address stigma and discrimination [16]. Social protection policies have also been recognized as essential to disease elimination strategies. For example, it is estimated that expanding social protection coverage could lead to a 9% annual reduction in TB incidence, with a cumulative 76.1% decrease by 2035 [17].

Considering the absence of standardized conceptual tools and process indicators in the Brazilian context that allow for an understanding of the operationalization and implementation of intersectoral actions and services related to social protection for TB control, there is a need to develop an instrument capable of evaluating the functioning of the social protection network for people affected by TB. Such an instrument would support planning and decision-making within and beyond the health sector, justifying the originality and relevance of this study, which is aligned with the goals of the WHO End TB Strategy, the UN SDGs, and the Brazilian National Plan for Ending TB.

Therefore, this study aimed to develop and validate an instrument composed of indicators for measuring and assessing the implementation of social protection actions for people affected by tuberculosis.

## Methods

### Study design

This was a methodological study aimed at developing and conducting content validation of an Evaluative Matrix by a panel of judges/experts. The study was carried out in the Brazilian context and organized into two systematic stages, conducted between March 2023 and May 2024.

### First stage: development of the logic model and evaluative matrix

Considering the measures and strategies aimed at ensuring social protection as a health intervention, the first step involved an in-depth study of each component of this intervention in relation to established standards and criteria, culminating in the use of a graphic resource commonly applied in normative evaluations, known as a Logic Model [18].

Thus, the first stage consisted of the construction and theoretical foundation of the research instrument for the elaboration of the Logic Model, which involved conducting a scoping review and a document analysis.

The scoping review analyzed the available evidence on measures and strategies considered sensitive and/or specific to TB, designed to guarantee social protection as a right for people affected by the disease [15]. Complementarily, the document analysis involved the collection of institutional technical materials, legislation, decrees, ordinances, resolutions, and other normative acts within the Brazilian context, at national, state, and municipal levels, produced by government bodies and their respective social control mechanisms.

From this process, four thematic categories emerged: the right to health, the right to social assistance, the right to social security, and the sharing of responsibilities [19].

Both studies informed the development of a representative and visual Logic Model of the articulation and operationalization of the measures and strategies recommended and/or implemented nationally, aiming to guarantee social protection as a right for people affected by TB.

**Fig. 1:**
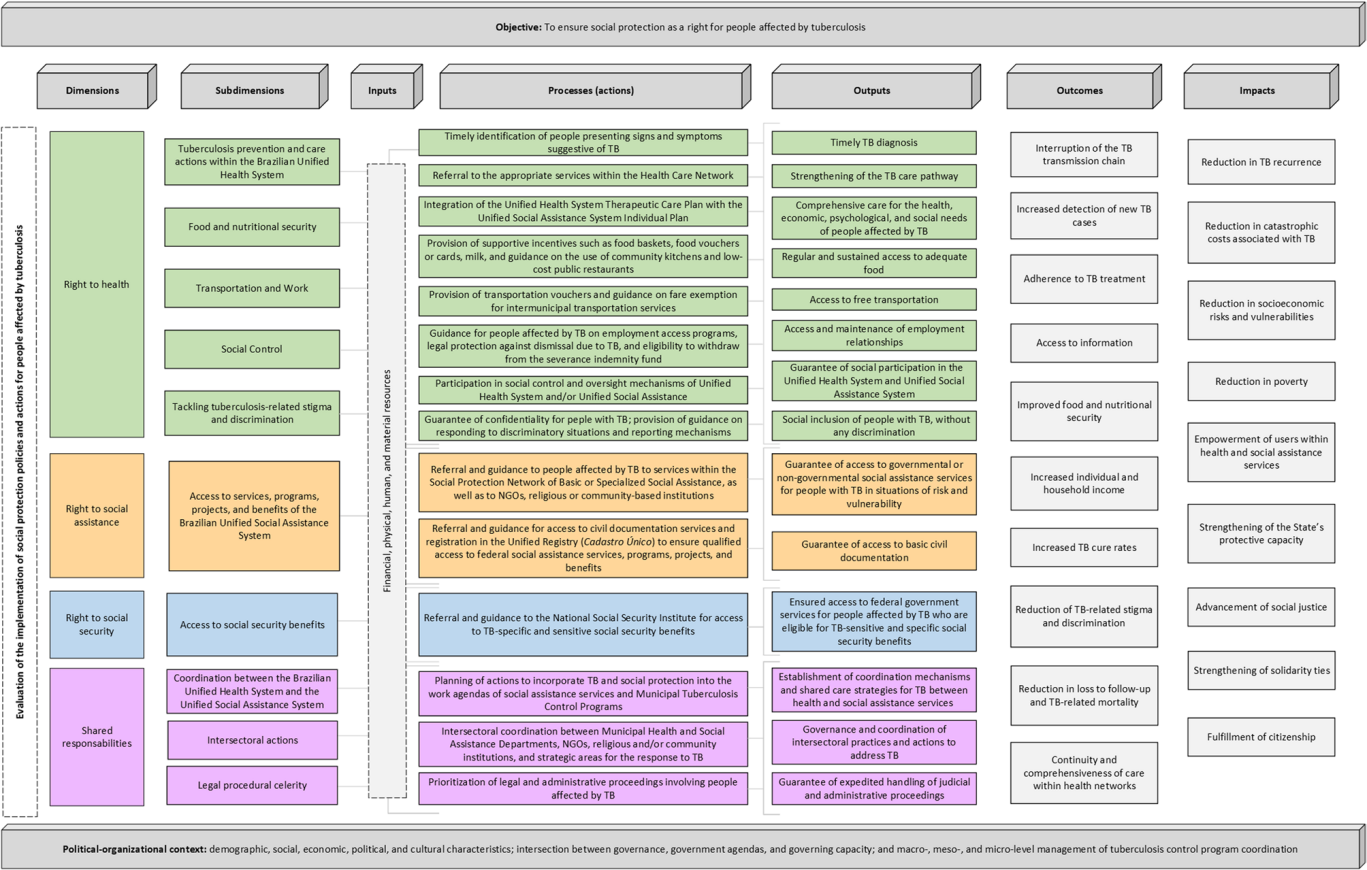
Logic model illustrating policies and strategies to guarantee social protection rights for people affected by tuberculosis Source: developed by the authors (2024)

The Logic Model was structured around the following elements: “dimensions”, “subdimensions”, “inputs”, “processes (actions)”, and the “outputs” generated from each dimension, as well as the resulting “outcomes” and “impacts” of the combined actions and outputs (Fig. 1).

The Logic Model supported the development of an instrument called the “Evaluative Matrix”, designed to assess the implementation of actions aimed at providing social protection for people affected by TB within the national context. This Matrix was composed of three axes from the Logic Model (“subdimensions”, “processes (actions)”, and “outputs”) and three additional axes based on each identified “output”, encompassing evaluative indicators, parameters, and data collection sources. It is noteworthy that the development of this instrument took place between October and December 2023.

### Second stage: validation of the evaluative matrix indicators

The second stage, concerning the content validation of the Evaluative Matrix, was initially conducted through meetings among members of the research group from the affiliated educational institution, in collaboration with an international partner, aiming to refine the instrument prior to consultation with experts. Subsequently, content validation was performed based on the analysis and evaluation by key informants (judges/experts) regarding the relevance, significance, and clarity of the set of indicators and related items.

Validation was conducted using the Delphi method, an innovative and systematic methodological approach that relies on the expert judgment to achieve consensus through multiple rounds of questionnaires, ensuring the anonymity of participants [20]. This method is considered particularly appropriate for the validation of quantitative and qualitative instruments in the health field [21].

The selection of judges for the expert panel was intentional and non-probabilistic, conducted between December 2023 and January 2024. It involved the review of curricula vitae available on the Lattes Platform, a national database that compiles academic and professional records of Brazilian researchers. The platform is widely used for identifying experts, assessing academic productivity, and supporting merit-based selection in science and academia. It is managed by the National Council for Scientific and Technological Development (*Conselho Nacional de Desenvolvimento Científico e Tecnológico* – CNPq), a federal government agency responsible for promoting scientific and technological research in Brazil through funding, scholarships, and evaluation mechanisms. For this study, the following search strategy was applied: simple search > “subject” – PhDs and other researchers > “nationality” – Brazilian > “search terms” – “Tuberculosis” and “Social Protection”.

Additionally, given that some curricula might not appear in the initial search, the selection process was expanded through manual searches of Brazilian authors of scientific publications on the topic and later supplemented using the “snowball” sampling method, where initially selected experts could nominate additional participants with similar profiles to join the study.

The expertise of potential judges was assessed based on inclusion criteria adapted from Fehring’s model [22], which assigns a maximum score of 14 points. A minimum score of five points was required for selection, based on the following: Master’s and/or Ph.D. degrees in health sciences or social and human sciences (mandatory criterion); a dissertation on TB and/or social protection (2 points); a thesis on TB and/or social protection (2 points); specialization in the area or related fields (1 point); development of research on TB and/or social protection (3 points); authorship of scientific articles on TB and/or social protection within the last five years (3 points); and at least two years of professional experience in the area or related fields (3 points). Candidates who did not achieve the minimum score, who did not respond to three consecutive invitations, or who failed to complete the instrument were excluded.

According to the literature, there is no ideal number of judges for the Delphi technique; decisions should consider costs, the nature of the problem, and the availability of specialists in the area [20]. Thus, after the initial search, 204 curricula were retrieved, with the first 100 analyzed consecutively via the Lattes Platform. An additional seven candidates were identified through manual searches, and two more through the snowball method. After applying the selection criteria, 41 judges were deemed eligible to participate.

Data collection was carried out via email, with an invitation letter explaining the study objectives and evaluation instructions. Upon agreeing to participate, key informants accessed a link to the electronic research instrument via the Google Forms^®^ platform.

The instrument included the Informed Consent Form (ICF), which participants were required to read and sign online before providing demographic data and proceeding to content validation (See in Supplementary file 1).

The pre-established deadline for returning the completed instrument was 20 days from receipt.

Judges rated each item using a four-point Likert scale, from 1 (strongly disagree) to 4 (strongly agree), with an open field for comments, suggestions, and/or proposed modifications. All evaluations were exported into a Microsoft Excel^®^ database and analyzed using descriptive statistics.

The content validity of the instrument was assessed using the Content Validity Index (CVI), calculated at both the item and the overall instrument levels. The CVI for each item (CVI-I) was determined by the proportion of experts who rated the item as either 3 (“agree”) or 4 (“strongly agree”) on a four-point Likert scale, according to the following formula:

$$\mathrm{CVI}-\mathrm I\;=\frac{\mathrm{Number}\;\mathrm{of}\;\mathrm{responses}\;\mathrm{rated}\;\mathrm{as}\;3\;\mathrm{or}\;4}{\mathrm{Total}\;\mathrm{number}\;\mathrm{of}\;\;\mathrm{respondents}}\;$$

Items with a CVI-I value lower than 0.80 were reviewed for revision or exclusion and subsequently resubmitted to the panel for re-evaluation in a subsequent Delphi round. Items with a CVI-I of 0.80 or higher were considered to have achieved satisfactory content validity.

The overall CVI (CVI-O) for the instrument was calculated by averaging the CVI-I values of all items, using the following formula:

$$\mathrm{CVI}-\mathrm O\;=\;\frac{\sum_{}\;\mathrm{CVI}-\mathrm I}{\mathrm{Total}\;\mathrm{numbers}\;\mathrm{of}\;\mathrm{items}}$$

An overall CVI of 0.80 or above was established as the threshold for adequate content validity of the final instrument [23].

### Ethics

The study was approved by the Research Ethics Committee of the Ribeirão Preto School of Nursing, University of São Paulo (approval number 6.389.278), and was conducted in accordance with Resolution nº 466/2012 of the Brazilian National Health Council, and the principles of the Declaration of Helsinki. All study participants received and signed the ICF, ensuring voluntary participation and anonymity. Furthermore, non-stigmatizing language related to TB was adopted throughout the entire study [24].

## Results

The methodological process of constructing the Evaluative Matrix enabled the mapping of the existing landscape of social protection for people affected by TB at the national level. The instrument was composed of 20 evaluative categories related to the “products” and 53 evaluative indicators, corresponding to the “processes (actions)” of the Logic Model, distributed across 11 subdimensions derived from the four main dimensions of the Logic Model: (I) the right to health; (II) the right to social assistance; (III) the right to social security; and (IV) the shared responsibilities (Table 1). For each indicator, parameters were established related to the type of data collection source, whether primary or secondary (Table 2).

**Table 1:** Number of evaluative categories and indicators for each subdimension and corresponding dimension in the evaluative matrix

Dimensions	Subdimensions	Evaluative categories	Evaluative indicators
Right to health	Tuberculosis prevention and care actions within the Brazilian Unified Health System	03	05
	Food and nutritional security	02	03
	Transportation	01	03
	Work	02	05
	Social control	01	02
	Tackling tuberculosis-related stigma and discrimination	01	04
Right to social assistance	Access to services, programs, projects, and benefits of the Brazilian Unified Social Assistance System	03	17
Right to social security	Access to social security benefits	04	06
Shared responsibilities	Coordination between the Brazilian Unified Health System and the Unified Social Assistance System	01	04
	Intersectoral actions	01	03
	Legal procedural celerity	01	01

Source: developed by the authors (2024)

**Table 2:** Evaluative Matrix of actions targeting social protection for people affected by tuberculosis, including dimensions, sub-dimensions, evaluation categories, indicators, parameters, and data sources

	**Subdimensions**	**Evaluation categories**	**Evaluation indicators**	Parameters*	**Data sources**
Right to Health	Tuberculosis prevention and care actions within the Brazilian Unified Health System	Timely tuberculosis diagnosis	Proportion of people diagnosed with tuberculosis by Primary Health Care teams and/or referral services	Satisfactory: ≥ 80% Regular: 60–79% Unsatisfactory: ≤ 59%	Tuberculosis information systems (secondary data)
		Strengthening of the tuberculosis care pathway	Access to tuberculosis care in health services	Satisfactory: 3.6 −5.0; Regular: 2.5–3.5; Unsatisfactory: 1.0–2.4	Interview with people affected by tuberculosis (primary data)
			Referrals to different services within the Health Care Network for tuberculosis care	Satisfactory: 3.6 −5.0; Regular: 2.5–3.5; Unsatisfactory: 1.0–2.4	Interview with people affected by tuberculosis (primary data)
			Tuberculosis prevention and health promotion activities in health services	Satisfactory: 3.6 −5.0; Regular: 2.5–3.5; Unsatisfactory: 1.0–2.4	Interview with health and social assistance professionals (primary data)
		Comprehensive care for the health, economic, psychological, and social needs of people affected by tuberculosis	Development of a Singular Therapeutic Project or other care planning strategies to ensure comprehensive care for people with tuberculosis and their families	Satisfactory: 3.6 −5.0; Regular: 2.5–3.5; Unsatisfactory: 1.0–2.4	Interview with health and social assistance professionals (primary data)
	Food and nutritional security	Guarantee of food provision	Provision of incentives such as (1) food baskets, (2) food vouchers or food cards, (3) milk	Satisfactory: 3.6 −5.0; Regular: 2.5–3.5; Unsatisfactory: 1.0–2.4	Interview with people affected by tuberculosis (primary data)
			Use of (1) popular restaurants and (2) community kitchens	Satisfactory: 3.6 −5.0; Regular: 2.5–3.5; Unsatisfactory: 1.0–2.4	Interview with people affected by tuberculosis (primary data)
		Regular and permanent access to food	Ability to have the main daily meals during tuberculosis treatment: (1) breakfast, (2) lunch, and (3) dinner	Satisfactory: 3.6 −5.0; Regular: 2.5–3.5; Unsatisfactory: 1.0–2.4	Interview with people affected by tuberculosis (primary data)
	Transportation	Access to free transportation	Exemption from intermunicipal transport fares	Satisfactory: 76–100%; Regular: 46–75%; Unsatisfactory: 0–45%	Interview with health professionals, social assistance professionals, and the Tuberculosis Control Program coordination (primary data)
			Provision of social transportation vouchers for companions of people with tuberculosis who are unable to move alone	Satisfactory: 76–100%; Regular: 46–75%; Unsatisfactory: 0–45%	Interview with health professionals, social assistance professionals, and the Tuberculosis Control Program coordination (primary data)
			Provision of bus transportation vouchers	Satisfactory: 3.6 −5.0; Regular: 2.5–3.5; Unsatisfactory: 1.0–2.4	Interview with people affected by tuberculosis (primary data)
	Work	Access to employment	Existence of programs facilitating access to the labor market	Satisfactory: 3.6 −5.0; Regular: 2.5–3.5; Unsatisfactory: 1.0–2.4	Interview with social assistance professionals (primary data)
		Maintenance of employment relationships	Prohibition of dismissal due to a tuberculosis diagnosis	Satisfactory: 3.6 −5.0; Regular: 2.5–3.5; Unsatisfactory: 1.0–2.4	Interview with health and social assistance professionals (primary data)
			Prohibition of dismissal due to a tuberculosis diagnosis	Satisfactory: ≥ 75% (yes); Unsatisfactory: ≤ 74% (no)	Interview with people affected by tuberculosis (primary data)
			Guarantee of access to the Severance Indemnity Fund for Employees (FGTS)	Satisfactory: 3.6 −5.0; Regular: 2.5–3.5; Unsatisfactory: 1.0–2.4	Interview with health and social assistance professionals (primary data)
			Guarantee of access to the Severance Indemnity Fund for Employees (FGTS)	Satisfactory: ≥ 75% (yes); Unsatisfactory: ≤ 74% (no)	Interview with people affected by tuberculosis (primary data)
	Social control	Guarantee of social participation in the Unified Health System (SUS) and Unified Social Assistance System (SUAS)	Participation of civil society, community representatives, and leaders in SUS and SUAS social control bodies to ensure the right to social protection for people affected by tuberculosis	Satisfactory: 3.6 −5.0; Regular: 2.5–3.5; Unsatisfactory: 1.0–2.4	Interview with health professionals, social assistance professionals, and the Tuberculosis Control Program coordination (primary data)
			Participation of civil society, community representatives, and leaders in SUS and SUAS social control bodies to ensure the right to social protection for people affected by tuberculosis	Satisfactory: 3.6 −5.0; Regular: 2.5–3.5; Unsatisfactory: 1.0–2.4	Interview with people affected by tuberculosis (primary data)
	Tackling tuberculosis-related stigma and discrimination	Social inclusion of people with tuberculosis within health and social assistance units, territories, and community spaces, without any inhumane or degrading treatment or discrimination due to their illness	Mandatory confidentiality regarding the condition of people with tuberculosis	Satisfactory: 3.6 −5.0; Regular: 2.5–3.5; Unsatisfactory: 1.0–2.4	Interview with health professionals, social assistance professionals, and the Tuberculosis Control Program coordination (primary data)
			Guidance on how to act in situations or attitudes of discrimination due to tuberculosis	Satisfactory: 3.6 −5.0; Regular: 2.5–3.5; Unsatisfactory: 1.0–2.4	Interview with people affected by tuberculosis (primary data)
			Information on communication channels and complaint services such as “*Disque 100”*, “*Disque 180”*, and “*Disque Saúde 136”*	Satisfactory: 3.6 −5.0; Regular: 2.5–3.5; Unsatisfactory: 1.0–2.4	Interview with health professionals, social assistance professionals, and the Tuberculosis Control Program coordination (primary data)
Right to Social Assistance	Access to services, programs, projects, and benefits of the Brazilian Unified Social Assistance System	Guarantee of access to governmental or non-governmental social assistance services for people with tuberculosis in situations of risk and vulnerability	Access to Basic Social Protection services – Reference Centers for Social Assistance (*CRAS*)	Satisfactory: 3.6 −5.0; Regular: 2.5–3.5; Unsatisfactory: 1.0–2.4	Interview with people affected by tuberculosis (primary data)
			Access to Specialized Social Protection services of medium complexity – (1) Specialized Reference Centers for Social Assistance and (*CREAS*) (2) Specialized Reference Centers for people experiencing homelessness (*Centro POP*)	Satisfactory: 3.6 −5.0; Regular: 2.5–3.5; Unsatisfactory: 1.0–2.4	Interview with people affected by tuberculosis (primary data)
			Access to Specialized Social Protection services of high complexity – Institutional or family shelter units	Satisfactory: 3.6 −5.0; Regular: 2.5–3.5; Unsatisfactory: 1.0–2.4	Interview with people affected by tuberculosis (primary data)
			Access to Social Workers within the Health and Social Assistance Networks	Satisfactory: ≥ 75% (yes); Unsatisfactory: ≤ 74% (no)	Interview with people affected by tuberculosis (primary data)
			Access to (1) non-governmental organizations; (2) religious institutions; (3) community institutions	Satisfactory: ≥ 75% (yes); Unsatisfactory: ≤ 74% (no)	Interview with people affected by tuberculosis (primary data)
			Referral of people with tuberculosis to the Social Assistance Network services	Satisfactory: 3.6 −5.0; Regular: 2.5–3.5; Unsatisfactory: 1.0–2.4	Interview with health and social assistance professionals (primary data)
		Guarantee of inclusion in the Single Registry (*CadÚnico*) for qualified offer of social assistance services, programs, projects, and benefits to people with tuberculosis in situations of risk and vulnerability	Recognition of people with tuberculosis registered in *CadÚnico* as an eligibility criterion for social assistance programs and services	Satisfactory: 3.6 −5.0; Regular: 2.5–3.5; Unsatisfactory: 1.0–2.4	Interview with social assistance professionals and social assistance management (primary data)
			Inclusion in *CadÚnico*	Satisfactory: ≥ 75% (yes); Unsatisfactory: ≤ 74% (no)	Interview with people affected by tuberculosis (primary data)
			Access to electricity tariff discounts	Satisfactory: ≥ 75% (yes); Unsatisfactory: ≤ 74% (no)	Interview with people affected by tuberculosis (primary data)
			Access to occasional social benefits	Satisfactory: ≥ 75% (yes); Unsatisfactory: ≤ 74% (no)	Interview with people affected by tuberculosis (primary data)
			Access to income transfer programs: (1) *Bolsa Família* Program; (2) Continuous Cash Benefit (*BPC*)	Satisfactory: ≥ 75% (yes); Unsatisfactory: ≤ 74% (no)	Interview with people affected by tuberculosis (primary data)
			Access to housing programs	Satisfactory: ≥ 75% (yes); Unsatisfactory: ≤ 74% (no)	Interview with people affected by tuberculosis (primary data)
			Access to other social benefits and/or programs: (1) *Bolsa Verde*; (2) Gas Aid; (3) Clothing acquisition programs	Satisfactory: ≥ 75% (yes); Unsatisfactory: ≤ 74% (no)	Interview with people affected by tuberculosis (primary data)
		Guarantee of access to basic civil documentation	Access to birth certificate/marriage certificate	Satisfactory: ≥ 75% (yes); Unsatisfactory: ≤ 74% (no)	Interview with people affected by tuberculosis (primary data)
			Access to personal identification documents (e.g., Identity Card, National Registry of Foreigners, Passport, Individual Taxpayer Registry, National Driver’s License)	Satisfactory: ≥ 75% (yes); Unsatisfactory: ≤ 74% (no)	Interview with people affected by tuberculosis (primary data)
			Access to the Work and Social Security Card	Satisfactory: ≥ 75% (yes); Unsatisfactory: ≤ 74% (no)	Interview with people affected by tuberculosis (primary data)
			Referral to the appropriate services for the issuance of civil documentation	Satisfactory: 3.6 −5.0; Regular: 2.5–3.5; Unsatisfactory: 1.0–2.4	Interview with health and social assistance professionals (primary data)
Right to Social Security	Access to social security benefits	Guarantee of access to federal services for people with tuberculosis eligible for social security benefits	Referral of people affected by tuberculosis to the National Institute of Social Security (*Instituto Nacional do Seguro Social - INSS*)	Satisfactory: 3.6 −5.0; Regular: 2.5–3.5; Unsatisfactory: 1.0–2.4	Interview with health and social assistance professionals (primary data)
			Access to tuberculosis-sensitive social security benefits: (1) maternity/paternity leave; (2) unemployment insurance; (3) incarceration allowance	Satisfactory: ≥ 75% (yes); Unsatisfactory: ≤ 74% (no)	Interview with people affected by tuberculosis (primary data)
		Waiver of the minimum contribution period for formally employed or self-employed workers who contribute to INSS when work incapacity is due to active tuberculosis	Granting of permanent disability retirement	Satisfactory: ≥ 75% (yes); Unsatisfactory: ≤ 74% (no)	Interview with people affected by tuberculosis (primary data)
			Granting of temporary disability benefit (Sick leave allowance)	Satisfactory: ≥ 75% (yes); Unsatisfactory: ≤ 74% (no)	Interview with people affected by tuberculosis (primary data)
		Income tax exemption on income from retirement, pension, or military reform, including supplementary income from private entities and alimony for people with active tuberculosis	Income tax exemption	Satisfactory: ≥ 75% (yes); Unsatisfactory: ≤ 74% (no)	Interview with people affected by tuberculosis (primary data)
		Possibility of withdrawing funds from the PIS/PASEP account by holders or dependents of people with active tuberculosis	Financial withdrawal from the PIS/PASEP fund	Satisfactory: ≥ 75% (yes); Unsatisfactory: ≤ 74% (no)	Interview with people affected by tuberculosis (primary data)
Shared responsibilities	Coordination between the Brazilian Unified Health System and the Unified Social Assistance System	Establishment of coordination mechanisms and shared care strategies for tuberculosis between social assistance services and the Health Care Network	Planning meetings between health and social assistance programs	Satisfactory: 3.6 −5.0; Regular: 2.5–3.5; Unsatisfactory: 1.0–2.4	Interview with health professionals, social assistance professionals, health and/or social assistance managers, and TB Program coordinators (primary data)
			Shared care for people with tuberculosis between health and social assistance services	Satisfactory: 3.6 −5.0; Regular: 2.5–3.5; Unsatisfactory: 1.0–2.4	Interview with health professionals, social assistance professionals, health and/or social assistance managers, and TB Program coordinators (primary data)
			Inclusion of tuberculosis in the social assistance work agenda	Satisfactory: 3.6 −5.0; Regular: 2.5–3.5; Unsatisfactory: 1.0–2.4	Interview with social assistance professionals and social assistance management (primary data)
			Inclusion of social protection in the work agenda of the Municipal Tuberculosis Control Program	Satisfactory: 3.6 −5.0; Regular: 2.5–3.5; Unsatisfactory: 1.0–2.4	Interview with health professionals, social assistance professionals, and the Tuberculosis Control Program coordination (primary data)
	Intersectoral actions	Management and organization of practices for implementing, monitoring, and evaluating intersectoral actions to address tuberculosis, especially among socially vulnerable populations	Coordination between the Municipal Department of Social Assistance and the Municipal Health Department to address tuberculosis	Satisfactory: 3.6 −5.0; Regular: 2.5–3.5; Unsatisfactory: 1.0–2.4	Interview with health and/or social assistance managers and the Tuberculosis Control Program coordination (primary data)
			Collaboration with NGOs, religious, and/or community-based institutions	Satisfactory: 3.6 −5.0; Regular: 2.5–3.5; Unsatisfactory: 1.0–2.4	Interview with health professionals, social assistance professionals, TB Program coordinators, representatives of the Municipal Health/Social Assistance Council, and service user council members (primary data)
			Coordination with strategic areas such as: (1) Primary Health Care, (2) Mental Health, (3) Occupational Health, (4) Prison System, (5) Street Outreach Teams, and (6) HIV/AIDS Program	Satisfactory: 3.6 −5.0; Regular: 2.5–3.5; Unsatisfactory: 1.0–2.4	Interview with health professionals, health managers, and the Tuberculosis Control Program coordination (primary data)
	Legal procedural celerity	Guarantee of expedited handling of judicial and administrative proceedings	Priority processing of judicial and administrative cases in which the person affected by tuberculosis is a party or has a vested interest	Satisfactory: ≥ 75% (yes); Unsatisfactory: ≤ 74% (no)	Interview with people affected by tuberculosis (primary data)

Source: developed by the authors (2024)

*Secondary data: Satisfactory: ≥ 80% (Primary Health Care and/or referral services), Regular: 60–79% (Primary Health Care and/or referral services), Unsatisfactory: ≤ 59% (Primary Health Care and/or referral services); Average Likert scale responses: Satisfactory: 3.6 to 5.0 (always/almost always), Regular: 2.5 to 3.5 (sometimes), Unsatisfactory: 1.0 to 2.4 (never/almost never); Yes/Sometimes/No responses: Satisfactory: 76–100% (yes); Regular: 46–75% (sometimes), Unsatisfactory: 0–45% (no); Yes/No responses: Satisfactory: ≥ 75% (yes), Unsatisfactory: ≤ 74% (no)

### Characterization of the panel of experts

In the process of reviewing and evaluating the Evaluative Matrix, the panel of experts was composed of nine judges/experts in the first round (Delphi I) and seven in the second round (Delphi II), with the loss of two judges due to failure to return the instrument. The minimum age of the group was 36 years and the maximum was 64 years (mean = 44.8 years), with a predominance of female participants (*n* = 8; 88.9%). In terms of racial diversity, participants self-identified as White (*n* = 4; 44.4%), Yellow (Asian) (*n* = 3; 33.3%), and Brown (Mixed race) (*n* = 2; 22.2%).

The majority of participants held a postdoctoral degree (*n* = 5; 55.6%). Regarding professional activities, the experts were mainly linked to teaching and research (*n* = 6; 66.7%), followed by representatives from civil society organizations (*n* = 2; 22.2%) and health management (*n* = 1; 11.1%). The Southeast region was the most represented geographically, with five participants, followed by the South and Central-West regions with two participants each, and the North region with one participant. No expert reported less than five years of experience in the field of TB and/or social protection.

### Validation of the evaluative matrix indicators

In the first Delphi round, the CVI-O was 0.96, indicating an excellent level of consensus among the judges regarding the set of indicators included in the Evaluative Matrix. However, five indicators obtained a CVI below 0.80. Of these, three did not achieve consensus regarding clarity, one regarding both clarity and relevance, and one regarding clarity, pertinence, and relevance (Table 3).

**Table 3:** Evaluation of five indicators for clarity, pertinence, and relevance in two Delphi rounds

Evaluative indicators		CVI – Delphi I	Interpretation	CVI – Delphi II	Interpretation
**Dimension – Right to Health**					
*Subdimension – Tuberculosis prevention and care actions within the Brazilian Unified Health System*					
Access to tuberculosis care services	Clarity	0.44	Not consensual for clarity	1.00	Consensual
	Pertinence	1.00		-	
	Relevance	1.00		-	
Prevention activities for tuberculosis illness and health promotion in health services	Clarity	0.67	Not consensual for clarity and pertinence	0.86	
	Pertinence	0.78		1.00	
	Relevance	0.89		-	
*Subdimension – Tackling tuberculosis-related stigma and discrimination*					
Obligation to preserve confidentiality regarding TB condition	Clarity	0.67	Not consensual for clarity	1.00	Consensual
	Pertinence	0.89		-	
	Relevance	0.89		-	
Guidance on conduct in situations of TB-related discrimination	Clarity	0.78	Not consensual for clarity	1.00	
	Pertinence	1.00		-	
	Relevance	1.00		-	
**Dimension – Right to Social Assistance**					
*Subdimension – Access to services*,* programs*,* projects*,* and benefits of the Brazilian Unified Social Assistance System*					
Recognition of people with tuberculosis registered in CadÚnico as an eligibility criterion for social assistance programs and services	Clarity	0.78	Not consensual for clarity, pertinence, and relevance	0.86	Consensual
	Pertinence	0.78		1.00	
	Relevance	0.78		1.00	

Source: developed by the authors (2024)

Regarding the suggested modifications, the feedback mainly focused on making the indicators objective, ensuring they effectively measured what they intended to assess; simple, expressing a single idea clearly; precise, with a clear distinction between items and avoiding confusion; and appropriate in terms of vocabulary, avoiding ambiguities.

Following the individual adjustment of each indicator based on the experts’ suggestions, a second Delphi round was conducted, during which all five indicators achieved a CVI greater than 0.80, thus reaching consensus without the need for a third evaluation round (Table 3). It is worth noting that, for each indicator that received feedback during Delphi rounds I and II, regardless of whether consensus was achieved, an individual discussion was held with the research team to refine the items for better understanding and agreement.

## Discussion

The set of evaluative categories and indicators reflects a systematic effort to encompass the various dimensions of the rights of people affected by TB, highlighting the complexity of social protection in addressing the disease. The Evaluative Matrix can be a valuable tool for planning and evaluating the social protection strategies implemented by TB service providers and other stakeholders. Its purpose is to accelerate progress toward eliminating TB as a public health problem by combating poverty and other social determinants, while protecting the human rights of people with TB and their families.

Although the End TB Strategy emphasizes the need to offer social interventions to people affected by TB, most WHO Member States, including Brazil, have not yet fully and sustainably incorporated social protection into their programmatic responses to the disease [1]. In this context, this study is pioneering in creating and validating an indicator-based instrument to evaluate the implementation of social protection actions for people affected by TB in Brazil.

It is important to clarify that the development of this instrument is not intended to justify the need for social protection, which is already recognized as a fundamental human right. Rather, the Evaluative Matrix serves as a methodological tool to assess how this right is being implemented in practice. By providing a structured approach to measure the reach, integration, and quality of social protection actions, the matrix contributes to the operationalization of rights and to identifying service gaps that hinder equitable access.

The development of this instrument also fosters an important reflection on the connection between the theoretical, legal, and institutional framework and the reality of the contexts in which the rights of people with TB may or may not be enforced. This is because vulnerabilities and social inequalities generate different levels of access to material and symbolic resources, impacting health and creating obstacles to the autonomy of people affected by the disease as citizens [25].

However, although the indicators are applicable to the Brazilian context, it is essential to consider the heterogeneous socioeconomic conditions of municipalities, given the disparities in access to health, education, income, and basic sanitation resources. Thus, the instrument can be adapted to different organizational and local health contexts, becoming a promising tool for understanding what services are being offered, how they are delivered, and what improvements can be made to ensure the social protection of people affected by TB.

Possible adaptations may include the prioritization of specific indicators according to local needs and vulnerabilities, linguistic and cultural adjustments, the simplification of data collection procedures in low-resource settings, or the integration of the matrix into existing local monitoring and evaluation tools.

In this methodological study, content validity was ensured by the quality of the expert panel, which included judges with extensive expertise in social protection and TB. The representation of almost all regions of the country also contributed to a broader assessment of the instrument, allowing the consideration of different local and regional perspectives, as well as their demographic, social, and economic characteristics.

Notably, the participation of two judges from Civil Society Organizations (CSO) enhanced the quality of the Matrix evaluation, as they represented advocates for the rights of people affected by TB. Although two judges did not participate in the second validation round, the number of experts involved was considered sufficient to ensure content validity in the health field [20].

Nevertheless, it is important to recognize the methodological challenges intrinsic to the Delphi technique, particularly the need for sustained engagement from participants across rounds, which may result in response fatigue and panel attrition. Additionally, the search for consensus can sometimes reduce the visibility of divergent perspectives [20]. To mitigate these risks, we adopted clear inclusion criteria for expert selection, maintained communication throughout the process, and ensured a diverse composition of the panel. These strategies contributed to maintaining participation, strengthening legitimacy, and ensuring diversity of perspectives in the final instrument.

Given the high overall CVI obtained in both rounds, exceeding the recommended minimum of 0.80 [23], it was concluded that the Matrix has excellent content validity. The contributions of the experts were essential for improving the clarity, relevance, and pertinence of the instrument’s items, particularly concerning key concepts used in the terminologies of the health, social assistance, and social security fields.

Regarding the evaluative categories and indicators that composed the subdimensions of the Right to Health, the process of constructing the Evaluative Matrix made it possible to identify elements reflecting the cross-cutting nature of this right and the broad concept of health established by the Brazilian Federal Constitution and the Organic Health Law [9]. This concept broadens the understanding of people affected by TB as rights holders, approaching them holistically.

Prevention and care actions for TB within the SUS, from a social protection perspective, must be oriented toward addressing social inequalities and health inequities that affect access to diagnosis, treatment, and various services within the health care network, as well as to the services provided by SUAS. Shared care is essential to strengthen the TB care continuum and, above all, to ensure comprehensive attention to the health, economic, psychological, and social needs of people with TB [26].

On the other hand, despite the universal and free TB treatment offered by SUS, studies have highlighted the economic and social impact of the disease, with catastrophic costs affecting 40.3% [27] and 41% [28] of families, reaching 48.1% of Brazilian households where expenses exceeded 20% of the annual income during treatment [29]. This impact is mainly due to unemployment or work interruption, as well as medical, transportation, and nutritional supplement costs [27–29].

In this context, the implementation of actions and strategies that promote food and nutritional security, along with expanded access to transportation and employment, is essential to address TB. This is justified by the fact that 89% of people with TB incur transportation costs, 56% incur costs with special food, and 60% lose their income due to work absenteeism during treatment [27].

Evaluative indicators focusing on these rights are fundamental to understanding how they are being implemented and operationalized. For instance, in settings where food vouchers are provided, TB treatment outcomes are significantly better, with a 13% higher cure rate (RR = 1.13, 95% CI: 1.03–1.21) in the intervention group compared to standard treatment [30].

Although there are legal and ethical regulations ensuring confidentiality regarding a person’s TB status in Brazil [31], a study revealed that 50% of unemployed people with TB remained unemployed due to the stigma associated with the disease [27], exacerbating the care trajectory and further marginalizing these people.

This reality highlights the importance of including evaluative indicators aimed at combating stigma and discrimination related to TB, thus reinforcing the right to non-humiliating, non-degrading treatment and the preservation of human dignity, while promoting a safer environment free from fear of unwanted disclosure.

Regarding the evaluative categories and indicators of the Subdimension of the Right to Social Assistance, during the validation of the Evaluative Matrix, experts recognized the importance of actions and strategies aimed at ensuring access to the SUAS in addressing TB. These actions directly relate to social rights and encompass the (re)production process of the disease, while also exposing potential barriers to the realization of these rights, such as the lack of access to basic civil documentation and difficulties linking to the Unified Registry, particularly for people with TB living in vulnerable and socially at-risk conditions.

Studies highlight the potential of income transfer programs within SUAS in improving TB treatment outcomes. In regions with high coverage of the BFP – Brazil’s largest conditional cash transfer program - there was a reduction in TB mortality rates [32] and disease incidence compared to areas with medium or low coverage [33].

Moreover, among people with TB enrolled in the BFP, there was an absolute increase in cure rates ranging from 7 to 11% [34], a 51% and 40% reduction in TB cases and mortality among the extremely poor, respectively, and a 63% decrease in incidence and a 65% decrease in mortality among indigenous populations, even though 56.2% of people with TB eligible for the benefit did not receive it [35]. Another study analyzing the effects of cash transfer programs through BFP and/or the *BPC* demonstrated an 8% improvement in cure rates among people undergoing TB treatment [36].

In relation to the evaluative categories of the Subdimension of the Right to Social Security, it was observed that access to social security benefits is conditioned by mandatory requirements, such as contributions to the Brazilian social security system, linked to formal employment. Although this requirement has a legal and constitutional basis, it can limit the coverage of social protection, as 60% of people affected by TB work in the informal sector [28].

A study revealed that before incurring TB-related costs, 42% of people affected by the disease were unemployed, a figure that rose to 71% after those costs emerged [27]. Furthermore, after the onset of respiratory symptoms, the daily working hours dropped from 6.5 h/day to 3.9 h/day, and 20% of people lost their jobs [37], worsening the economic impact on affected families.

In this scenario, it becomes evident that poorer families, heavily affected by TB, face major difficulties accessing social security rights. Many of these people lack formal employment contracts and continue to work informally even when sick, hindering treatment adherence [28]. For those with formal employment, ensuring social security rights is crucial to mitigating the negative impacts of TB, especially in cases of permanent disability.

However, a study showed that even with formal employment and work incapacity, the right to sick leave benefits was not always granted [38]. It is worth noting that active TB is listed under Brazilian legislation as a disease exempt from qualifying periods for social security benefits, allowing individuals without prior contributions to access benefits even without formal employment history [39].

Regarding the evaluative categories and indicators that composed the subdimensions of Shared Responsibilities, the validation process of the Evaluative Matrix highlighted the importance of intersectoral coordination between health and social assistance services as a key pillar in addressing TB. Shared care, combined with the strengthening of social protection and support networks, not only broadens access to treatment but also provides essential support for overcoming the vulnerable conditions that exacerbate the health-disease process, influenced by social determinants [25].

Another important aspect concerns indicators related to partnerships with non-governmental organizations (NGOs), religious institutions, and community organizations, which have the potential to offer a broader environment of care and social support. Moreover, integration with strategic areas such as Primary Care, mental health services, the prison system, and programs like Street Clinics plays a crucial role in ensuring continuity of care for people affected by TB, particularly those with comorbidities, those experiencing homelessness, or with a history of incarceration [26].

Ensuring the prioritization of judicial and administrative processes involving people with TB represents significant progress in protecting their rights [39]. Prioritizing these cases reflects recognition of individuals’ health conditions and social vulnerabilities, enabling faster and more effective responses from the Brazilian justice system.

Nevertheless, it is necessary to emphasize the urgency of equipping social actors directly or indirectly impacted by TB to fully exercise their rights. This includes training and empowering individuals regarding their health, social security, and social rights, which must be strengthened and safeguarded by the State, family, and society. This process can contribute to improving public policies and social protection, expanding access for people in situations of vulnerability and social risk, and strengthening the fight for equity in addressing TB [38].

It is important to highlight that some strategies present in the Evaluative Matrix are focused on TB treatment and characterized as “specific TB actions”, directly benefiting people with TB and their families when incorporated into existing TB programs. Others are part of a broader social protection framework, with the potential to modify the structural conditions of society [1, 40]. However, this study focused on the construction and validation of an Evaluative Matrix and did not investigate the outcomes of social protection actions and policies on the lives of people affected by TB.

### Limitations

The study has some limitations. The expert panel was composed predominantly of professionals from the Southeast Region of Brazil, which may limit the geographic representativeness of the findings, particularly for regions such as the Northeast and North, where social vulnerability profiles and TB epidemiology may differ. Although the Northeast was included in the initial sample of invited experts, no potential participants from this region responded to the invitation. Future studies should include broader regional representation to capture diverse territorial realities and policy implementation contexts.

The absence of people directly affected by TB in the validation process is also a limitation, as their lived experiences could have enriched the evaluative dimensions with user-centered perspectives. Additionally, the attrition of two panelists between Delphi rounds, although minimal, may have influenced the diversity of viewpoints.

While the high CVI values indicate strong internal content validity, the Evaluative Matrix has not yet undergone external validation in real-world implementation settings. Future research is needed to test its operational feasibility and effectiveness across different social, institutional, and health system contexts.

Another important limitation concerns the instrument’s complexity and length, which may present challenges for its application in municipalities with limited technical capacity or resources. In such contexts, the use of tailored adaptations and local capacity-building strategies will be essential to support its effective implementation.

Furthermore, it is important to consider that the population affected by TB in Brazil is highly heterogeneous. Groups such as people experiencing homelessness, people deprived of liberty, indigenous people, and others in situations of extreme vulnerability may have more limited access to social protection in all evaluated dimensions. These structural inequalities should be acknowledged in the application and interpretation of the matrix results, and future versions of the instrument may benefit from further refinements that take these differences into account.

These limitations, however, do not detract from the value of the instrument as a foundational tool to guide public policies and strengthen social protection for people affected by TB in Brazil.

## Conclusions

The results of this study demonstrated that the proposed Evaluative Matrix was validated with satisfactory CVI values, indicating significant consensus among the experts. The final validated version of the Matrix consists of four dimensions, 20 evaluative categories, and 53 indicators, along with their respective parameters and data collection sources. These components provide a solid foundation to support future evaluations of the implementation of social protection actions targeting people affected by TB.

The development of the logical model and the Evaluative Matrix was grounded in a well-defined and robust theoretical and methodological framework, resulting in an innovative evaluation tool to assist managers and policymakers in the formulation and improvement of such policies. In addition to its strategic relevance, the Matrix is notable for its flexibility, allowing for adaptations to various local contexts.

## Supplementary Information

Supplementary Material 1.

## Data Availability

Data is provided within the manuscript or supplementary information file. The results from the online questionnaire during this Delphi study are available from the corresponding author upon reasonable request.

## Abbreviations

BFP: *Programa Bolsa Família* (Bolsa Família Program)
BPC: *Benefício de Prestação Continuada* (Continuous Cash Benefit)
CadÚnico: *Cadastro Único* (Unified Registry)
Centro POP: *Centro de Referência Especializado para Pessoas em Situação de Rua* (Specialized Reference Centers for people experiencing homelessness)
CNPq: *Conselho Nacional de Desenvolvimento Científico e Tecnológico* (Brazilian National Council for Scientific and Technological Development
CRAS: *Centros de Referência em Assistência Social* (Reference Centers for Social Assistance)
CREAS: *Centros de Referência Especializados de Assistência Social* (Specialized Reference Centers for Social Assistance)
CSO: Civil Society Organizations
CIEDDS: Committee for the Elimination of Tuberculosis and Other Socially Determined Diseases
CVI: Content Validity Index
CVI-I: Content Validity Index for each item
CVI-O: Overall Content Validity Index
FGTS: Fundo de Garantia do Tempo de Serviço (Guarantee of access to the Severance Indemnity Fund for Employees)
ICF: Informed Consent Form
INSS: *Instituto Nacional do Seguro Social* (National Institute of Social Security)
LOAS: *Lei Orgânica da Assistência Social *(Organic Law of Social Assistance)
NGOs: Non-Governmental Organizations
SDGs: Sustainable Development Goals
SUAS: *Sistema Único de Assistência Social *(Unified Social Assistance System)
SUS: *Sistema Único de Saúde *(Unified Health System)
TB: Tuberculosis
UN: United Nations
WHO: World Health Organization
