# Supplementary material for: Evaluation of social protection for people affected by tuberculosis: development of an indicator matrix for Brazil
Source: BMC Public Health. 2025 Nov 25;25:4127. doi: 10.1186/s12889-025-24689-7 (PMC12645765; doi:10.1186/s12889-025-24689-7)
Supplement: Supplementary file 1 — Supplementary Material 1. [file 12889_2025_24689_MOESM1_ESM.pdf]

Analysis and Judgment Matrix –Assessment of Social Protection for People Affected by Tuberculosis

INFORMED CONSENT FORM

Dear Participant,

We would like to invite you to take part in a research study entitled "Assessment of social protection for people affected by tuberculosis". This study is being conducted by researcher *Melisane Regina Lima Ferreira*, a graduate student at the Ribeirão Preto School of Nursing, University of São Paulo, under the supervision of Professor *Dr. Aline Aparecida Monroe*, a faculty member at the same institution.

Study objective: To assess social protection provided to people affected by tuberculosis in the municipality of Ribeirão Preto, São Paulo.

Procedures: You will be directed to an online data collection platform where you may participate in this study by completing a questionnaire, which is expected to take approximately one hour and twenty minutes. The first part of the questionnaire will ask for basic identification information, and the second part will include: 1) An evaluation of the clarity, relevance, and importance of the actions described in the Evaluation Matrix, using a Likert-type scale (strongly disagree, disagree, agree, strongly agree), with an open field for your comments and suggestions; and 2) An assessment of the matrix in terms of semantics, clarity of the questions, writing, and coherence between the items of each component, with open-ended responses.
You may withdraw from this study at any time without the need to provide justification, and your decision will not affect your professional work or standing within your institution. You will not incur any costs, nor will you receive any financial compensation for participating. If you choose to participate, a downloadable version of this Informed Consent Form will be made available to you, and it is recommended that you keep a copy for your records.

Benefits: This study aims to support health and social care professionals and policymakers in understanding people affected by tuberculosis in a broader social context, thereby strengthening the responsiveness of health services in coordination with the social protection network. The data and results of this study will be disseminated in academic settings as well as through public communication channels.

Risks: The risks associated with this study relate to potential difficulty in responding to the evaluation instrument. If you encounter any such difficulties, the researcher may be contacted to provide clarification. Participants may also experience fatigue; in which case they may pause and resume the evaluation as needed.

Confidentiality: Your identity will be kept confidential. All completed questionnaires will be anonymized by replacing your name with a code. Please note that you have the right to compensation under applicable national legislation should any harm result from your participation in this study.

This project has been approved by the Research Ethics Committee of the Ribeirão Preto School of Nursing – USP (CEP-EERP/USP), which ensures ethical protection of research participants, under opinion number 6.232.046. Should you have any questions about research ethics, you may contact CEP-EERP/USP at:
Phone: +55 (16) 3315-9197
Email: cep@eerp.usp.br
Address: Av. dos Bandeirantes, 3900 - Ribeirão Preto, SP, Brazil
Office hours: Monday to Friday, from 10:00 to 12:00 and from 14:00 to 16:00.

Thank you for your collaboration.

Prof. Dr. Aline Aparecida Monroe (amonroe@eerp.usp.br)
Melisane Regina Lima Ferreira (melisanerlf@usp.br)
Contact phone: +55 (16) 3315-3407

*Required question

1. Email *

2. In light of the guidelines previously presented, I agree* to participate in this research by signing this consent form. Thus, I consent to participate in the research project in question.

To complete the form, please reserve approximately **1 hour and 20 minutes of your time**, or fill it out gradually/on different days using the registered email, as your responses will be saved. Kindly observe the established deadline of 15 business days for submission.

Select only one option.

( ) Yes ( ) No

Identification Data

In this first part of the questionnaire, you will answer questions about your identification data so we can understand the profile of the specialists participating in this study.

3. What is your age (in years)? *

____

4. What gender do you identify with? *

Select only one option.

- ◯ Cisgender woman
- ◯ Transgender woman
- ◯ Cisgender man
- ◯ Transgender man
- ◯ Non-binary
- ◯ Prefer not to say

5. What is your self-declared race/ethnicity? *

Select only one option.

- ◯ Black
- ◯ Brown (Pardo)
- ◯ White
- ◯ Yellow (Asian descent)
- ◯ Indigenous
- ◯ Prefer not to say

6. What is your highest academic degree? *

Select only one option.

- ◯ Undergraduate degree
- ◯ Specialization
- ◯ Master's degree
- ◯ Doctorate (PhD)
- ◯ Post-doctorate

7. Which of the following categories do you belong to? *

Select only one option.

- ◯ Tuberculosis Control Program Coordination
- ◯ Health Management
- ◯ Social Assistance Management
- ◯ Teaching/Research
- ◯ Health Professionals
- ◯ Social Assistance Professionals
- ◯ Civil Society Representatives
- ◯ Other: _______________________________

8. In which region of the country do you work? *

Select only one option.

- ( ) North ( ) Northeast ( ) Central-West ( ) Southeast ( ) South

9. How long have you worked with tuberculosis or social protection issues? *

Select only one option.

- ( ) Less than 3 years ( ) 3 to 5 years ( ) 5 to 10 years ( ) More than 10 years

Assessment of the Set of Indicators in the Evaluation Matrix

From this point forward, you will evaluate the **indicators** of the Evaluation Matrix regarding the clarity, relevance, and pertinence of the items that comprise each indicator - that is, the **measures**, **parameters**, **data collection sources**, and the **calculation method or guiding question**. To do so, you will use a Likert-type scale (**strongly disagree, disagree, agree, strongly agree**), accompanied by an open-ended field where you may provide suggestions regarding semantics, question clarity, wording, and coherence among the items within each indicator.

The instrument is composed of 20 evaluation indicators and 50 measures, which are distributed across 11 subdimensions, grouped into the following dimensions: I) Right to Health; II) Right to Social Assistance; III) Right to Social Security; and IV) Shared Responsibilities.

Dimension I – Right to Health

This first dimension, related to the **Right to Health**, includes 6 (six) subdimensions, totaling 10 (ten) indicators and 19 (nineteen) measures.

Subdimension: Tuberculosis Prevention and Care Actions within the Unified Health System (*Sistema Único de Saúde - SUS*)

10. **Indicator 1:** Timely Tuberculosis Diagnosis*

Measure 1: Timely identification of people presenting signs and symptoms of tuberculosis by health teams in Primary Health Care and/or specialized reference services

Parameter:

Satisfactory: ≥ 80% (Primary Health Care and/or reference services)

Fair: 60% to 79% (Primary Health Care and/or reference services)

Unsatisfactory: ≤ 59% (Primary Health Care and/or reference services)

Data source: TB-WEB (secondary data from the state of São Paulo)

Calculation method:

Number of tuberculosis cases reported in Primary Health Care and in specialized reference services (***timely diagnosis***) / Total number of tuberculosis cases reported in the municipality of Ribeirão Preto in 2023–2024

Please mark only one option per row.

	Strongly disagree	Disagree	Agree	Strongly agree
Clarity	⬭	⬭	⬭	⬭
Pertinence	⬭	⬭	⬭	⬭
Relevance	⬭	⬭	⬭	⬭

11. Please use the space below to provide your comments, criticisms, or suggestions regarding the evaluated indicator, particularly in terms of semantics, question clarity, wording, and coherence among its components.

12. **Indicator 2:** Strengthening the Tuberculosis Care Continuum*

Measure 1: Access to tuberculosis care in health services

Parameter: Likert Scale:
Satisfactory: 3.6 to 5.0 (always/almost always)
Fair: 2.5 to 3.5 (sometimes)
Unsatisfactory: 1.0 to 2.4 (never/almost never)
Data source: Interviews with people affected by tuberculosis (primary data)
Questions:
1) Did you have any difficulty accessing a tuberculosis diagnosis?

Do you have access to the following services during your tuberculosis treatment?:

a) Tuberculosis medication

b) Monthly follow-up consultations for tuberculosis treatment

c) Directly Observed Treatment (DOT) for tuberculosis

d) Home visits during tuberculosis treatment

e) Vaccination

f) Evaluation of close contacts for tuberculosis diagnosis

Please mark only one option per row.

	Strongly disagree	Disagree	Agree	Strongly agree
Clarity	◯	◯	◯	◯
Pertinence	◯	◯	◯	◯
Relevance	◯	◯	◯	◯

13. Please use the space below to provide your comments, criticisms, or suggestions regarding the evaluated indicator, particularly in terms of semantics, question clarity, wording, and coherence among its components.

14. **Indicator 2:** Strengthening the Tuberculosis Care Continuum*
Measure 2: Referrals made to different services within the Health Care Network for tuberculosis care
Parameter: Likert Scale:
Satisfactory: 3.6 to 5.0 (always/almost always)
Fair: 2.5 to 3.5 (sometimes)
Unsatisfactory: 1.0 to 2.4 (never/almost never)
Data source: Interviews with people affected by tuberculosis (primary data)
Question: *When you have another health issue, are you able to get referred to another health service?*

Please mark only one option per row.

	Strongly disagree	Disagree	Agree	Strongly agree
Clarity	○	○	○	○
Pertinence	○	○	○	○
Relevance	○	○	○	○

15. Please use the space below to provide your comments, criticisms, or suggestions regarding the evaluated indicator, particularly in terms of semantics, question clarity, wording, and coherence among its components.

16. **Indicator 2:** Strengthening the Tuberculosis Care Continuum*
Measure 3: Tuberculosis prevention and health promotion activities carried out in health services
Parameter: Likert Scale:
Satisfactory: 3.6 to 5.0 (always/almost always)
Fair: 2.5 to 3.5 (sometimes)
Unsatisfactory: 1.0 to 2.4 (never/almost never)
Data source: Interviews with health and social care professionals (primary data)
Question: *Are tuberculosis prevention and health promotion activities carried out in this health service?*

Please mark only one option per row.

	Strongly disagree	Disagree	Agree	Strongly agree
Clarity				
Pertinence				
Relevance				

17. Please use the space below to provide your comments, criticisms, or suggestions regarding the evaluated indicator, particularly in terms of semantics, question clarity, wording, and coherence among its components.

18. **Indicator 3:** Comprehensive care for the health, economic, psychological, and social needs of people affected by tuberculosis*

Measure 1: Coordination between the Individual Therapeutic Project (*Projeto Terapêutico Singular – PTS*) of the Unified Health System (SUS) and the Individual Assistance Plan (*Plano Individual de Atendimento*) of the Unified Social Assistance System (SUAS) to ensure comprehensive care for the person with tuberculosis and their family

Parameter: Likert Scale:

Satisfactory: 3.6 to 5.0 (always/almost always)

Fair: 2.5 to 3.5 (sometimes)

Unsatisfactory: 1.0 to 2.4 (never/almost never)

Data source: Interviews with health and social care professionals (primary data)

Question: *Is there coordination between the Individual Therapeutic Project of SUS and the Individual Assistance Plan of SUAS to ensure comprehensive care for the person with tuberculosis and their family?*

Please mark only one option per row.

	Strongly disagree	Disagree	Agree	Strongly agree
Clarity	⬭	⬭	⬭	⬭
Pertinence	⬭	⬭	⬭	⬭
Relevance	⬭	⬭	⬭	⬭

19. Please use the space below to provide your comments, criticisms, or suggestions regarding the evaluated indicator, particularly in terms of semantics, question clarity, wording, and coherence among its components.

Subdimension: Food and Nutritional Security

20. **Indicator 1:** Food Provision*

Measure 1: Provision of benefits such as (1) food baskets, (2) food vouchers or food cards
Parameter: Likert Scale:
Satisfactory: 3.6 to 5.0 (always/almost always)
Fair: 2.5 to 3.5 (sometimes)
Unsatisfactory: 1.0 to 2.4 (never/almost never)
Data source: Interviews with people affected by tuberculosis (primary data)
Questions:
1) Food baskets
Are you currently receiving food baskets because you are undergoing tuberculosis treatment?

2) Food voucher or food card

Are you currently receiving a food voucher because you are undergoing tuberculosis treatment?

Please mark only one option per row.

	Strongly disagree	Disagree	Agree	Strongly agree
Clarity	⬭	⬭	⬭	⬭
Pertinence	⬭	⬭	⬭	⬭
Relevance	⬭	⬭	⬭	⬭

21. Please use the space below to provide your comments, criticisms, or suggestions regarding the evaluated indicator, particularly in terms of semantics, question clarity, wording, and coherence among its components.

22. **Indicator 1:** Food Provision*
Measure 2: Use of (1) public restaurants and (2) community kitchens
Parameter: Likert Scale:
Satisfactory: 3.6 to 5.0 (always/almost always)
Fair: 2.5 to 3.5 (sometimes)
Unsatisfactory: 1.0 to 2.4 (never/almost never)
Data source: Interviews with people affected by tuberculosis (primary data)
Question: *Are you currently enrolled in any program to access food, such as: (1) public restaurants; (2) community kitchens?*

Please mark only one option per row.

	Strongly disagree	Disagree	Agree	Strongly agree
Clarity	◯	◯	◯	◯
Pertinence	◯	◯	◯	◯
Relevance	◯	◯	◯	◯

23. Please use the space below to provide your comments, criticisms, or suggestions regarding the evaluated indicator, particularly in terms of semantics, question clarity, wording, and coherence among its components.

24. **Indicator 2:** Regular and Continuous Access to Food*
Measure 1: Ability to have the three main meals during the day: (1) breakfast, (2) lunch, and (3) dinner
Parameter: Likert Scale:
Satisfactory: 3.6 to 5.0 (always/almost always)
Fair: 2.5 to 3.5 (sometimes)
Unsatisfactory: 1.0 to 2.4 (never/almost never)
Data source: Interviews with people affected by tuberculosis (primary data)
Question: *Are you currently able to have the three main meals during the day: (1) Breakfast; (2) Lunch; (3) Dinner?*

Please mark only one option per row.

	Strongly disagree	Disagree	Agree	Strongly agree
Clarity				
Pertinence				
Relevance				

25. Please use the space below to provide your comments, criticisms, or suggestions regarding the evaluated indicator, particularly in terms of semantics, question clarity, wording, and coherence among its components.

Subdimension: Transportation

26. **Indicator 1:** Access to Free Transportation*

Measure 1: Fare exemption for intermunicipal transportation services

Parameter: Yes / Sometimes / No:

Satisfactory: 76 to 100% (yes)

Fair: 46 to 75% (sometimes)

Unsatisfactory: 0 to 45% (no)

Data source: Interviews with health professionals, social assistance professionals, and tuberculosis control program coordinators (primary data)

Question: *In your municipality, is there a fare exemption for intermunicipal transportation services for people undergoing tuberculosis treatment?*

Please mark only one option per row.

	Strongly disagree	Disagree	Agree	Strongly agree
Clarity				
Pertinence				
Relevance				

27. Please use the space below to provide your comments, criticisms, or suggestions regarding the evaluated indicator, particularly in terms of semantics, question clarity, wording, and coherence among its components.

28. **Indicator 1:** Access to Free Transportation*
Measure 2: Provision of a transportation voucher (*vale social*) for the companion of a person with tuberculosis who is unable to move independently or cannot travel unaccompanied
Parameter: Yes / Sometimes / No:
Satisfactory: 76 to 100% (yes)
Fair: 46 to 75% (sometimes)
Unsatisfactory: 0 to 45% (no)
Data source: Interviews with health professionals, social assistance professionals, and tuberculosis control program coordinators (primary data)
Question: *In the municipality, is a transportation voucher (vale social) provided to the companion of a person with tuberculosis who is unable to move independently or cannot travel unaccompanied?*

Please mark only one option per row.

	Strongly disagree	Disagree	Agree	Strongly agree
Clarity	⬭	⬭	⬭	⬭
Pertinence	⬭	⬭	⬭	⬭
Relevance	⬭	⬭	⬭	⬭

29. Please use the space below to provide your comments, criticisms, or suggestions regarding the evaluated indicator, particularly in terms of semantics, question clarity, wording, and coherence among its components.

30. **Indicator 1:** Access to Free Transportation*
Measure 3: Provision of bus transportation vouchers
Parameter: Likert Scale:
Satisfactory: 3.6 to 5.0 (always/almost always)
Fair: 2.5 to 3.5 (sometimes)
Unsatisfactory: 1.0 to 2.4 (never/almost never)
Data source: Interviews with people affected by tuberculosis (primary data)
Question: *Are you currently receiving transportation vouchers because you are undergoing tuberculosis treatment?*

Please mark only one option per row.

	Strongly disagree	Disagree	Agree	Strongly agree
Clarity				
Pertinence				
Relevance				

31. Please use the space below to provide your comments, criticisms, or suggestions regarding the evaluated indicator, particularly in terms of semantics, question clarity, wording, and coherence among its components.

Subdimension: Work

32. **Indicator 1:** Access to the Labor Market*
Measure 1: Existence of programs that provide access to the labor market
Parameter: Likert Scale:
Satisfactory: 3.6 to 5.0 (always/almost always)
Fair: 2.5 to 3.5 (sometimes)
Unsatisfactory: 1.0 to 2.4 (never/almost never)
Data source: Interviews with social assistance professionals and tuberculosis control program coordinators (primary data)
Question: *Are people undergoing tuberculosis treatment informed by health services about the existence of programs that provide access to the labor market?*

Please mark only one option per row.

	Strongly disagree	Disagree	Agree	Strongly agree
Clarity				
Pertinence				
Relevance				

33. Please use the space below to provide your comments, criticisms, or suggestions regarding the evaluated indicator, particularly in terms of semantics, question clarity, wording, and coherence among its components.

34. **Indicator 2:** Maintenance of Employment Relationships*

Measure 1: Prohibition of dismissal from work due to contracting tuberculosis

Parameter 1: Likert Scale:

Satisfactory: 3.6 to 5.0 (always/almost always)

Fair: 2.5 to 3.5 (sometimes)

Unsatisfactory: 1.0 to 2.4 (never/almost never)

Data source: Interviews with social assistance professionals and tuberculosis control program coordinators (primary data)

Question: *Are people undergoing tuberculosis treatment informed by health services about the legal prohibition of dismissal from work due to having contracted tuberculosis?*

Parameter 2: Yes / No

Satisfactory: ≥ 75% (no)

Unsatisfactory: ≤ 74% (yes)

Data source: Interviews with people affected by tuberculosis (primary data)

Question: *Has your tuberculosis illness caused any unpaid leave or dismissal from your job (formal, self-employed, or informal)?*

Calculation method:

Number of people with tuberculosis who were dismissed or placed on leave due to tuberculosis during treatment / Number of people with tuberculosis under follow-up

Please mark only one option per row.

Strongly disagree	Disagree	Agree	Strongly agree

Clarity	⬭	⬭	⬭	⬭
Pertinence	⬭	⬭	⬭	⬭
Relevance	⬭	⬭	⬭	⬭

35. Please use the space below to provide your comments, criticisms, or suggestions regarding the evaluated indicator, particularly in terms of semantics, question clarity, wording, and coherence among its components.

36. **Indicator 2:** Maintenance of Employment Relationships*

Measure 2: Guarantee of access to the Severance Indemnity Fund for Employees (FGTS)

Parameter 1: Likert Scale:

Satisfactory: 3.6 to 5.0 (always/almost always)

Fair: 2.5 to 3.5 (sometimes)

Unsatisfactory: 1.0 to 2.4 (never/almost never)

Data source: Interviews with social assistance professionals and tuberculosis control program coordinators (primary data)

Question: *Are people undergoing tuberculosis treatment informed by health services about the possibility of withdrawing their FGTS due to illness?*

Parameter 2: Yes / No

Satisfactory: ≥ 75% (no)

Unsatisfactory: ≤ 74% (yes)

Data source: Interviews with people affected by tuberculosis (primary data)

Question: *Were you able to withdraw your FGTS early due to tuberculosis?*

Calculation method:

Number of people with tuberculosis who were able to withdraw their FGTS early during treatment / Number of people with tuberculosis under follow-up

Please mark only one option per row.

	Strongly disagree	Disagree	Agree	Strongly agree
Clarity				
Pertinence				
Relevance				

37. Please use the space below to provide your comments, criticisms, or suggestions regarding the evaluated indicator, particularly in terms of semantics, question clarity, wording, and coherence among its components.

Subdimension: Social Control

38. **Indicator 1:** Guarantee of Social Participation in the Unified Health System (SUS) and Unified Social Assistance System (SUAS)*

Measure 1: Participation of civil society, community representatives, and local leaders in SUS and SUAS social control bodies to ensure the right to social protection for people affected by tuberculosis

Parameter 1: Likert Scale:

Satisfactory: 3.6 to 5.0 (always/almost always)

Fair: 2.5 to 3.5 (sometimes)

Unsatisfactory: 1.0 to 2.4 (never/almost never)

Data source: Interviews with health professionals, social assistance professionals, and tuberculosis control program coordinators (primary data)

Question: *Is there participation from civil society, representatives, and community leaders in the social control bodies of SUS and SUAS to ensure the right to social protection for people affected by tuberculosis?*

Parameter 2: Likert Scale:

Satisfactory: 3.6 to 5.0 (always/almost always)

Fair: 2.5 to 3.5 (sometimes)

Unsatisfactory: 1.0 to 2.4 (never/almost never)

Data source: Interviews with people affected by tuberculosis (primary data)

Question: *Have you ever participated in a health council or committee to discuss tuberculosis-related issues within SUS or SUAS?*

Please mark only one option per row.

	Strongly disagree	Disagree	Agree	Strongly agree
Clarity				
Pertinence				
Relevance				

39. Please use the space below to provide your comments, criticisms, or suggestions regarding the evaluated indicator, particularly in terms of semantics, question clarity, wording, and coherence among its components.

40. **Indicator 1:** Guarantee of Social Participation in the Unified Health System (SUS) and Unified Social Assistance System (SUAS)*

Measure 2: Existence of forums/committees for coordination between social assistance and health focused on tuberculosis

Parameter: Likert Scale:

Satisfactory: 3.6 to 5.0 (always/almost always)

Fair: 2.5 to 3.5 (sometimes)

Unsatisfactory: 1.0 to 2.4 (never/almost never)

Data source: Interviews with health professionals, social assistance professionals, and tuberculosis control program coordinators (primary data)

Question: *Are meetings held through forums or committees for coordination between social assistance and health focused on tuberculosis?*

Please mark only one option per row.

	Strongly disagree	Disagree	Agree	Strongly agree
Clarity				
Pertinence				
Relevance				

41. Please use the space below to provide your comments, criticisms, or suggestions regarding the evaluated indicator, particularly in terms of semantics, question clarity, wording, and coherence among its components.

Subdimension: Combating Stigma and Discrimination of Tuberculosis

42. **Indicator 1:** Social inclusion of people with tuberculosis within health and social assistance units, in the territory and community spaces, without any form of inhumane or degrading treatment or discrimination due to their morbidity*

Measure 1: Mandatory confidentiality regarding the condition of the person with TB

Parameter: Likert Scale:

Satisfactory: 3.6 to 5.0 (always/almost always)

Fair: 2.5 to 3.5 (sometimes)

Unsatisfactory: 1.0 to 2.4 (never/almost never)

Data source: Interviews with health professionals, social assistance professionals, and tuberculosis control program coordinators (primary data)

Question: *Are people with tuberculosis in treatment informed by health service professionals about the mandatory confidentiality regarding their condition?*

Please mark only one option per row.

	Strongly disagree	Disagree	Agree	Strongly agree
Clarity				
Pertinence				
Relevance				

43. Please use the space below to provide your comments, criticisms, or suggestions regarding the evaluated indicator, particularly in terms of semantics, question clarity, wording, and coherence among its components.

44. **Indicator 1:** Social inclusion of people with tuberculosis within health and social assistance units, in the territory and community spaces, without any form of inhumane or degrading treatment or discrimination due to their morbidity*

Measure 2: Guidance on how to respond to discriminatory situations or attitudes due to tuberculosis

Parameter 1: Likert Scale:

Satisfactory: 3.6 to 5.0 (always/almost always)

Fair: 2.5 to 3.5 (sometimes)

Unsatisfactory: 1.0 to 2.4 (never/almost never)

Data source: Interviews with health and social assistance professionals (primary data)

Question: *Is it routine to provide guidance to people undergoing tuberculosis treatment on how to respond to situations or attitudes of discrimination due to the disease?*

Parameter 2: Likert Scale:

Satisfactory: 3.6 to 5.0 (always/almost always)

Fair: 2.5 to 3.5 (sometimes)

Unsatisfactory: 1.0 to 2.4 (never/almost never)

Data source: Interviews with people affected by tuberculosis (primary data)

Question: *Have you ever been guided on what to do if you experience a discriminatory situation or attitude because of tuberculosis?*

Please mark only one option per row.

	Strongly disagree	Disagree	Agree	Strongly agree
Clarity	◯	◯	◯	◯
Pertinence	◯	◯	◯	◯
Relevance	◯	◯	◯	◯

45. Please use the space below to provide your comments, criticisms, or suggestions regarding the evaluated indicator, particularly in terms of semantics, question clarity, wording, and coherence among its components.

____

____

____

____

____

46. **Indicator 1:** Social inclusion of people with tuberculosis within health and social assistance units, in the territory and community spaces, without any form of inhumane or degrading treatment or discrimination due to their morbidity*

Measure 3: Guidance on the existence of communication and complaint reporting channels through ombudsman offices or services such as "Disque 100", "Disque 180", and "Disque Saúde 136"

Parameter 1: Likert Scale:

Satisfactory: 3.6 to 5.0 (always/almost always)

Fair: 2.5 to 3.5 (sometimes)

Unsatisfactory: 1.0 to 2.4 (never/almost never)

Data source: Interviews with health professionals, social assistance professionals, and tuberculosis control program coordinators (primary data)

Question: *Are people undergoing tuberculosis treatment informed in health services about the existence of communication and complaint reporting channels through ombudsman offices or services such as "Disque 100", "Disque 180", and "Disque Saúde 136"?*

Please mark only one option per row.

	Strongly disagree	Disagree	Agree	Strongly agree
Clarity	◯	◯	◯	◯
Pertinence	◯	◯	◯	◯
Relevance	◯	◯	◯	◯

47. Please use the space below to provide your comments, criticisms, or suggestions regarding the evaluated indicator, particularly in terms of semantics, question clarity, wording, and coherence among its components.

Dimension II – Right to Social Assistance

This second dimension, referring to the Right to Social Assistance, includes only one subdimension, with a total of **3 (three) indicators** and **17 (seventeen) measures**.

Subdimension: Access to services, programs, projects, and social assistance benefits within the Unified Social Assistance System (SUAS)

48. **Indicator 1:** Guarantee of access to governmental or non-governmental social assistance services for people with tuberculosis in situations of risk and vulnerability*

Measure 1: Access to Basic Social Protection social assistance services – Social Assistance Reference Centers (CRAS)

Parameter: Likert Scale:

Satisfactory: 3.6 to 5.0 (always/almost always)

Fair: 2.5 to 3.5 (sometimes)

Unsatisfactory: 1.0 to 2.4 (never/almost never)

Data source: Interviews with people affected by tuberculosis (primary data)

Question: *Do you currently have access to social assistance through the Social Assistance Reference Centers (CRAS)?*

Please mark only one option per row.

Strongly disagree	Disagree	Agree	Strongly agree

Clarity	⬭	⬭	⬭	⬭
Pertinence	⬭	⬭	⬭	⬭
Relevance	⬭	⬭	⬭	⬭

49. Please use the space below to provide your comments, criticisms, or suggestions regarding the evaluated indicator, particularly in terms of semantics, question clarity, wording, and coherence among its components.

____

____

____

____

____

50. **Indicator 1:** Guarantee of access to governmental or non-governmental social assistance services for people with tuberculosis in situations of risk and vulnerability*

Measure 2: Access to Specialized Social Protection social assistance services of medium complexity – (1) Specialized Social Assistance Reference Centers (CREAS) and (2) Specialized Reference Center for Homeless People (Centro POP)

Parameter: Likert Scale:

Satisfactory: 3.6 to 5.0 (always/almost always)

Fair: 2.5 to 3.5 (sometimes)

Unsatisfactory: 1.0 to 2.4 (never/almost never)

Data source: Interviews with people affected by tuberculosis (primary data)

Questions:

1) *Do you currently have access to social assistance through the Specialized Social Assistance Reference Centers (CREAS)?*

2) *Do you currently have access to social assistance through the Specialized Reference Center for Homeless People (Centro POP)?*

Please mark only one option per row.

	Strongly disagree	Disagree	Agree	Strongly agree

Clarity	⬭	⬭	⬭	⬭
Pertinence	⬭	⬭	⬭	⬭
Relevance	⬭	⬭	⬭	⬭

51. Please use the space below to provide your comments, criticisms, or suggestions regarding the evaluated indicator, particularly in terms of semantics, question clarity, wording, and coherence among its components.

52. **Indicator 1:** Guarantee of access to governmental or non-governmental social assistance services for people with tuberculosis in situations of risk and vulnerability*

Measure 3: Access to high-complexity Specialized Social Protection social assistance services – Institutional or family shelter units

Parameter: Likert Scale:

Satisfactory: 3.6 to 5.0 (always/almost always)

Fair: 2.5 to 3.5 (sometimes)

Unsatisfactory: 1.0 to 2.4 (never/almost never)

Data source: Interviews with people affected by tuberculosis (primary data)

Question: *Do you currently have access to social assistance through institutional or family shelter units?*

Please mark only one option per row.

	Strongly disagree	Disagree	Agree	Strongly agree
Clarity	⬭	⬭	⬭	⬭
Pertinence	⬭	⬭	⬭	⬭
Relevance	⬭	⬭	⬭	⬭

53. Please use the space below to provide your comments, criticisms, or suggestions regarding the evaluated indicator, particularly in terms of semantics, question clarity, wording, and coherence among its components.

54. **Indicator 1:** Guarantee of access to governmental or non-governmental social assistance services for people with tuberculosis in situations of risk and vulnerability*

Measure 4: Access to Social Workers in the Health Care and Social Assistance Network

Parameter: Yes/ No

Satisfactory: ≥ 75% (yes)

Unsatisfactory: ≤ 74% (no)

Data source: Interviews with people affected by tuberculosis (primary data)

Question: *Did you receive any social assistance during your tuberculosis treatment?*

Calculation method: Number of people with tuberculosis who have access to social workers in the Health Care and/or Social Assistance Network / Number of people with tuberculosis under follow-up

Please mark only one option per row.

	Strongly disagree	Disagree	Agree	Strongly agree
Clarity				
Pertinence				
Relevance				

55. Please use the space below to provide your comments, criticisms, or suggestions regarding the evaluated indicator, particularly in terms of semantics, question clarity, wording, and coherence among its components.

56. **Indicator 1:** Guarantee of access to governmental or non-governmental social assistance services for people with tuberculosis in situations of risk and vulnerability*

Measure 5: Access to (1) Non-Governmental Organizations (NGOs); (2) Religious institutions; (3) Community organizations

Parameter: Yes/ No

Satisfactory: ≥ 75% (yes)

Unsatisfactory: ≤ 74% (no)

Data source: Interviews with people affected by tuberculosis (primary data)

Question: *Do you currently receive any social or financial assistance through (1) NGOs, (2) religious institutions, or (3) community organizations?*

Calculation method: Number of people with tuberculosis who have access to (1), (2), or (3) / Number of people with tuberculosis under follow-up

Please mark only one option per row.

	Strongly disagree	Disagree	Agree	Strongly agree
Clarity	⬭	⬭	⬭	⬭
Pertinence	⬭	⬭	⬭	⬭
Relevance	⬭	⬭	⬭	⬭

57. Please use the space below to provide your comments, criticisms, or suggestions regarding the evaluated indicator, particularly in terms of semantics, question clarity, wording, and coherence among its components.

58. **Indicator 1:** Guarantee of access to governmental or non-governmental social assistance services for people with tuberculosis in situations of risk and vulnerability*

Measure 6: Referral of people affected by tuberculosis to the Social Assistance Care Network services

Parameter: Likert Scale:

Satisfactory: 3.6 to 5.0 (always/almost always)

Fair: 2.5 to 3.5 (sometimes)

Unsatisfactory: 1.0 to 2.4 (never/almost never)

Data source: Interviews with health and social assistance professionals (primary data)

Question: *Is it part of the routine to refer people affected by tuberculosis to the Social Assistance Care Network services?*

Please mark only one option per row.

	Strongly disagree	Disagree	Agree	Strongly agree
Clarity	⬭	⬭	⬭	⬭
Pertinence	⬭	⬭	⬭	⬭
Relevance	⬭	⬭	⬭	⬭

59. Please use the space below to provide your comments, criticisms, or suggestions regarding the evaluated indicator, particularly in terms of semantics, question clarity, wording, and coherence among its components.

____

____

____

____

____

60. **Indicator 2:** Guarantee of inclusion in the Unified Registry (CadÚnico) for the qualified provision of federal government social assistance services, programs, projects, and benefits to people with tuberculosis in situations of risk and vulnerability*

Measure 1: Recognition of people with tuberculosis registered in CadÚnico as an eligibility criterion for social assistance programs and services

Parameter: Likert Scale:

Satisfactory: 3.6 to 5.0 (always/almost always)

Fair: 2.5 to 3.5 (sometimes)

Unsatisfactory: 1.0 to 2.4 (never/almost never)

Data source: Interviews with health professionals, social assistance professionals, and social assistance managers (primary data)

Question: *Is tuberculosis recognized as a vulnerability factor among people registered in CadÚnico for access to social assistance programs and services?*

Please mark only one option per row.

	Strongly disagree	Disagree	Agree	Strongly agree
Clarity	⬭	⬭	⬭	⬭
Pertinence	⬭	⬭	⬭	⬭
Relevance	⬭	⬭	⬭	⬭

61. Please use the space below to provide your comments, criticisms, or suggestions regarding the evaluated indicator, particularly in terms of semantics, question clarity, wording, and coherence among its components.

62. **Indicator 2:** Guarantee of inclusion in the Unified Registry (CadÚnico) for the qualified provision of federal government social assistance services, programs, projects, and benefits to people with tuberculosis in situations of risk and vulnerability*

Measure 2: Inclusion in the Unified Registry (CadÚnico)

Parameter: Yes/ No

Satisfactory: ≥ 75% (yes)

Unsatisfactory: ≤ 74% (no)

Data source: Interviews with people affected by tuberculosis (primary data)

Question: *Are you registered in the social assistance system, CadÚnico?*

Calculation method: Number of people with tuberculosis registered in CadÚnico / Number of people with tuberculosis under follow-up

Please mark only one option per row.

	Strongly disagree	Disagree	Agree	Strongly agree
Clarity				
Pertinence				
Relevance				

63. Please use the space below to provide your comments, criticisms, or suggestions regarding the evaluated indicator, particularly in terms of semantics, question clarity, wording, and coherence among its components.

64. **Indicator 2:** Guarantee of inclusion in the Unified Registry (CadÚnico) for the qualified provision of federal government social assistance services, programs, projects, and benefits to people with tuberculosis in situations of risk and vulnerability*

Measure 3: Access to discount on electricity bill
Parameter: Yes/ No

Satisfactory: ≥ 75% (yes)

Unsatisfactory: ≤ 74% (no)

Data source: Interviews with people affected by tuberculosis (primary data)

Question: *Do you currently have access to the Social Electricity Tariff??*

Calculation method: Number of people with tuberculosis who receive a discount on their electricity bill/ number of people with tuberculosis under follow-up who are registered in CadÚnico

Please mark only one option per row.

	Strongly disagree	Disagree	Agree	Strongly agree
Clarity				
Pertinence				
Relevance				

65. Please use the space below to provide your comments, criticisms, or suggestions regarding the evaluated indicator, particularly in terms of semantics, question clarity, wording, and coherence among its components.

66. **Indicator 2:** Guarantee of inclusion in the Unified Registry (CadÚnico) for the qualified provision of federal government social assistance services, programs, projects, and benefits to people with tuberculosis in situations of risk and vulnerability*

Measure 4: Access to emergency social benefits
Parameter: Yes/ No

Satisfactory: ≥ 75% (yes)

Unsatisfactory: ≤ 74% (no)

Data source: Interviews with people affected by tuberculosis (primary data)

Question: *Do you currently receive any of these benefits due to an event or situation you are experiencing? For example: maternity benefit, funeral assistance, aid in situations of temporary vulnerability, or assistance in cases of disasters and public calamities.*

Calculation method: Number of people with tuberculosis who receive emergency social benefits / Number of people with tuberculosis under follow-up who are registered in CadÚnico

Please mark only one option per row.

	Strongly disagree	Disagree	Agree	Strongly agree
Clarity	○	○	○	○
Pertinence	○	○	○	○
Relevance	○	○	○	○

67. Please use the space below to provide your comments, criticisms, or suggestions regarding the evaluated indicator, particularly in terms of semantics, question clarity, wording, and coherence among its components.

____

____

____

____

____

68. **Indicator 2:** Guarantee of inclusion in the Unified Registry (CadÚnico) for the qualified provision of federal government social assistance services, programs, projects, and benefits to people with tuberculosis in situations of risk and vulnerability*

Measure 5: Access to income transfer programs: (1) Bolsa Família Program; (2) Continuous Cash Benefit (BPC)

Parameter: Yes/ No

Satisfactory: ≥ 75% (yes)

Unsatisfactory: ≤ 74% (no)

Data source: Interviews with people affected by tuberculosis (primary data)

Question: *Do you currently participate in any of the following income transfer programs: (1) Bolsa Família Program (PBF); (2) Continuous Cash Benefit (BPC)?*

Calculation method: Number of people with tuberculosis who receive (1) PBF and/or (2) BPC / Number of people with tuberculosis under follow-up who are registered in CadÚnico

Please mark only one option per row.

	Strongly disagree	Disagree	Agree	Strongly agree
Clarity	⬭	⬭	⬭	⬭
Pertinence	⬭	⬭	⬭	⬭
Relevance	⬭	⬭	⬭	⬭

69. Please use the space below to provide your comments, criticisms, or suggestions regarding the evaluated indicator, particularly in terms of semantics, question clarity, wording, and coherence among its components.

70. **Indicator 2:** Guarantee of inclusion in the Unified Registry (CadÚnico) for the qualified provision of federal government social assistance services, programs, projects, and benefits to people with tuberculosis in situations of risk and vulnerability*

Measure 6: Access to Housing Programs
Parameter: Yes/ No

Satisfactory: ≥ 75% (yes)

Unsatisfactory: ≤ 74% (no)

Data source: Interviews with people affected by tuberculosis (primary data)

Question: *Do you currently participate in any housing program?*

Calculation method: Number of people with tuberculosis who have access to a Housing Program / Number of people with tuberculosis under follow-up who are registered in CadÚnico

Please mark only one option per row.

	Strongly disagree	Disagree	Agree	Strongly agree
Clarity				
Pertinence				
Relevance				

71. Please use the space below to provide your comments, criticisms, or suggestions regarding the evaluated indicator, particularly in terms of semantics, question clarity, wording, and coherence among its components.

72. **Indicator 2:** Guarantee of inclusion in the Unified Registry (CadÚnico) for the qualified provision of federal government social assistance services, programs, projects, and benefits to people with tuberculosis in situations of risk and vulnerability*

Measure 7: Access to other social assistance benefits and/or programs: (1) Bolsa Verde; (2) Gas Assistance; (3) Clothing Acquisition Program

Parameter: Yes/ No

Satisfactory: ≥ 75% (yes)

Unsatisfactory: ≤ 74% (no)

Data source: Interviews with people affected by tuberculosis (primary data)

Question: *Do you currently receive Bolsa Verde, Gas Assistance, or participate in any program to receive clothing while undergoing tuberculosis treatment?*

Calculation method: Number of people with tuberculosis who receive (1), (2), or (3) / Number of people with tuberculosis under follow-up who are registered in CadÚnico

Please mark only one option per row.

	Strongly disagree	Disagree	Agree	Strongly agree
Clarity				
Pertinence				
Relevance				

73. Please use the space below to provide your comments, criticisms, or suggestions regarding the evaluated indicator, particularly in terms of semantics, question clarity, wording, and coherence among its components.

74. **Indicator 3:** Guarantee of access to basic civil documentation*

Measure 1: Access to a Birth Certificate / Marriage Certificate or Certificate of Stable Union

Parameter: Yes/ No

Satisfactory: ≥ 75% (yes)

Unsatisfactory: ≤ 74% (no)

Data source: Interviews with people affected by tuberculosis (primary data)

Question: *Do you have a birth certificate and/or a marriage or stable union certificate?*

Calculation method: Number of people with tuberculosis who have a birth certificate and/or a marriage or stable union certificate / Number of people with tuberculosis under follow-up

Please mark only one option per row.

	Strongly disagree	Disagree	Agree	Strongly agree
Clarity				
Pertinence				
Relevance				

75. Please use the space below to provide your comments, criticisms, or suggestions regarding the evaluated indicator, particularly in terms of semantics, question clarity, wording, and coherence among its components.

76. **Indicator 3:** Guarantee of access to basic civil documentation*

Measure 2: Access to General Registration (RG) / National Registry of Foreigners (RNE) / Passport / Individual Taxpayer Registry (CPF) / National Driver's License (CNH)

Parameter: Yes/ No

Satisfactory: ≥ 75% (yes)

Unsatisfactory: ≤ 74% (no)

Data source: Interviews with people affected by tuberculosis (primary data)

Question: *Do you have an RG, RNE, Passport, CPF, or CNH?*

Calculation method: Number of people with tuberculosis who have an RG, RNE, Passport, CPF, or CNH / Number of people with tuberculosis under follow-up

Please mark only one option per row.

	Strongly disagree	Disagree	Agree	Strongly agree
Clarity				
Pertinence				
Relevance				

77. Please use the space below to provide your comments, criticisms, or suggestions regarding the evaluated indicator, particularly in terms of semantics, question clarity, wording, and coherence among its components.

78. **Indicator 3:** Guarantee of access to basic civil documentation*

Measure 3: Access to the Work and Social Security Card (Carteira de Trabalho e Previdência Social – CTPS)

Parameter: Yes/ No

Satisfactory: ≥ 75% (yes)

Unsatisfactory: ≤ 74% (no)

Data source: Interviews with people affected by tuberculosis (primary data)

Question: *Do you have a Work and Social Security Card (Carteira de Trabalho e Previdência Social)?*

Calculation method: Number of people with tuberculosis who have a Work and Social Security Card / Number of people with tuberculosis under follow-up

Please mark only one option per row.

	Strongly disagree	Disagree	Agree	Strongly agree
Clarity	⬭	⬭	⬭	⬭
Pertinence	⬭	⬭	⬭	⬭
Relevance	⬭	⬭	⬭	⬭

79. Please use the space below to provide your comments, criticisms, or suggestions regarding the evaluated indicator, particularly in terms of semantics, question clarity, wording, and coherence among its components.

80. **Indicator 3:** Guarantee of access to basic civil documentation*

Measure 4: Referral to the appropriate services for obtaining civil documentation

Parameter: Likert Scale:

Satisfactory: 3.6 to 5.0 (always/almost always)

Fair: 2.5 to 3.5 (sometimes)

Unsatisfactory: 1.0 to 2.4 (never/almost never)

Data source: Interviews with health and social assistance professionals (primary data)

Question: *In cases where a person with tuberculosis does not have any civil documentation, is it part of the service routine to refer them to the appropriate services to obtain their documents?*

Please mark only one option per row.

	Strongly disagree	Disagree	Agree	Strongly agree
Clarity				
Pertinence				
Relevance				

81. Please use the space below to provide your comments, criticisms, or suggestions regarding the evaluated indicator, particularly in terms of semantics, question clarity, wording, and coherence among its components.

Dimension III – Right to Social Security

This third dimension, concerning the Right to Social Security, includes only one subdimension, with a total of **04 (four) indicators** and **06 (six) measures**.

Subdimension: Access to Social Security Benefits

82. **Indicator 1:** Guarantee of access to federal government services by people with tuberculosis eligible to receive social security benefits*

Measure 1: Referral of people affected by tuberculosis to the National Institute of Social Security (INSS) for access to social security benefits

Parameter: Likert Scale:

Satisfactory: 3.6 to 5.0 (always/almost always)

Fair: 2.5 to 3.5 (sometimes)

Unsatisfactory: 1.0 to 2.4 (never/almost never)

Data source: Interviews with health and social assistance professionals (primary data)

Question: *Is it part of the routine to refer people with tuberculosis to social security benefits when work incapacity is due to active tuberculosis?*

Please mark only one option per row.

Strongly disagree	Disagree	Agree	Strongly agree

Clarity	⬭	⬭	⬭	⬭
Pertinence	⬭	⬭	⬭	⬭
Relevance	⬭	⬭	⬭	⬭

83. Please use the space below to provide your comments, criticisms, or suggestions regarding the evaluated indicator, particularly in terms of semantics, question clarity, wording, and coherence among its components.

____

____

____

____

____

84. **Indicator 1:** Guarantee of access to federal government services by people with tuberculosis eligible to receive social security benefits*

Measure 2: Access to social security benefits sensitive to tuberculosis: (1) Maternity/paternity leave; (2) Unemployment insurance; (3) Incarceration aid

Parameter: Yes/No

Satisfactory: ≥ 75% (yes)

Unsatisfactory: ≤ 74% (no)

Data source: Interviews with people affected by tuberculosis (primary data)

Questions:

1) *Do you currently receive maternity/paternity leave?*

2) *Do you currently receive unemployment insurance?*

3) *Do you currently receive incarceration aid because a family member is deprived of liberty?*

For the denominator: *Do you make any payment to contribute to social security?*

Calculation method: Number of people with tuberculosis who receive (1), (2), or (3) / Number of people with tuberculosis under follow-up who contribute to social security

Please mark only one option per row.

	Strongly disagree	Disagree	Agree	Strongly agree

Clarity	◯	◯	◯	◯
Pertinence	◯	◯	◯	◯
Relevance	◯	◯	◯	◯

85. Please use the space below to provide your comments, criticisms, or suggestions regarding the evaluated indicator, particularly in terms of semantics, question clarity, wording, and coherence among its components.

86. **Indicator 2:** Guarantee of exemption from the qualifying period for workers with formal employment or self-employed who contribute to the INSS when the work incapacity is due to active tuberculosis*

Measure 1: Granting of permanent disability retirement ("invalidity")

Parameter: Yes/No

Satisfactory: ≥ 75% (yes)

Unsatisfactory: ≤ 74% (no)

Data source: Interviews with people affected by tuberculosis (primary data)

Question: *Do you currently receive permanent disability retirement due to tuberculosis?*

For the denominator: *Do you make any payment to contribute to social security?*

Calculation method: Number of people with tuberculosis who receive permanent disability retirement / Number of people with tuberculosis under follow-up and contributing to social security

Please mark only one option per row.

	Strongly disagree	Disagree	Agree	Strongly agree
Clarity	◯	◯	◯	◯
Pertinence	◯	◯	◯	◯
Relevance	◯	◯	◯	◯

87. Please use the space below to provide your comments, criticisms, or suggestions regarding the evaluated indicator, particularly in terms of semantics, question clarity, wording, and coherence among its components.

____

____

____

____

____

88. **Indicator 2:** Guarantee of exemption from the qualifying period for workers with formal employment or self-employed who contribute to the INSS when the work incapacity is due to active tuberculosis*

Measure 2: Granting of temporary disability benefit (“sickness benefit”)

Parameter: Yes/No

Satisfactory: ≥ 75% (yes)

Unsatisfactory: ≤ 74% (no)

Data source: Interviews with people affected by tuberculosis (primary data)

Question: *Do you currently receive sickness benefit due to tuberculosis?*

For the denominator: *Do you make any payment to contribute to social security?*

Calculation method: Number of people with tuberculosis who receive sickness benefit / Number of people with tuberculosis under follow-up and contributing to social security

Please mark only one option per row.

	Strongly disagree	Disagree	Agree	Strongly agree
Clarity	⬭	⬭	⬭	⬭
Pertinence	⬭	⬭	⬭	⬭
Relevance	⬭	⬭	⬭	⬭

89. Please use the space below to provide your comments, criticisms, or suggestions regarding the evaluated indicator, particularly in terms of semantics, question clarity, wording, and coherence among its components.

90. **Indicator 3:** Guarantee of exemption on income related to retirement, pension, or reform, including the complement received from private entities and alimony for people affected by active tuberculosis*

Measure 1: Income tax exemption
Parameter: Yes/No

Satisfactory: ≥ 75% (yes)

Unsatisfactory: ≤ 74% (no)

Data source: Interviews with people affected by tuberculosis (primary data)

Question: *Do you currently have income tax exemption due to being under treatment for tuberculosis?*

For the denominator: *Do you file your income tax return? (for those with income compatible with filing a tax return)*

Calculation method: Number of people with tuberculosis who have income tax exemption due to illness / Number of people with tuberculosis under follow-up who file income tax returns

Please mark only one option per row.

	Strongly disagree	Disagree	Agree	Strongly agree
Clarity	⬭	⬭	⬭	⬭
Pertinence	⬭	⬭	⬭	⬭
Relevance	⬭	⬭	⬭	⬭

91. Please use the space below to provide your comments, criticisms, or suggestions regarding the evaluated indicator, particularly in terms of semantics, question clarity, wording, and coherence among its components.

92. **Indicator 4:** Possibility of accessing the PIS/PASEP account by account holders or dependents of people with active tuberculosis*

Measure 1: Financial withdrawal from PIS/PASEP

Parameter: Yes/No

Satisfactory: ≥ 75% (yes)

Unsatisfactory: ≤ 74% (no)

Data source: Interviews with people affected by tuberculosis (primary data)

Question: *Have you currently managed to withdraw your PIS/PASEP funds due to being under treatment for tuberculosis?*

For the denominator: *Do you make any contributions to social security?*

Calculation method: Number of people with tuberculosis who withdrew PIS/PASEP funds / Number of people with tuberculosis under follow-up and contributing to social security

Please mark only one option per row.

	Strongly disagree	Disagree	Agree	Strongly agree
Clarity	◯	◯	◯	◯
Pertinence	◯	◯	◯	◯
Relevance	◯	◯	◯	◯

93. Please use the space below to provide your comments, criticisms, or suggestions regarding the evaluated indicator, particularly in terms of semantics, question clarity, wording, and coherence among its components.

Dimension IV – Shared Responsibilities

This fourth and final dimension, related to the Shared Responsibilities, includes three (3) subdimensions, with a total of **three (3) indicators** and **eight (8) measures**.

Subdimension: Coordination between the Unified Social Assistance System (SUAS) and the Unified Health System (SUS)

94. **Indicator 1:** Establishment of coordination mechanisms and shared care for tuberculosis between social assistance services and services of the Health Care Network*

Measure 1: Joint action planning meetings between health and social assistance programs

Parameter: Likert Scale:

Satisfactory: 3.6 to 5.0 (always/almost always)

Fair: 2.5 to 3.5 (sometimes)

Unsatisfactory: 1.0 to 2.4 (never/almost never)

Data source: Interviews with health professionals, social assistance professionals, health and/or social assistance managers, and coordinators of the Tuberculosis Control Program (primary data)

Question: *How often are joint action planning meetings held between health and social assistance programs?*

Please mark only one option per row.

	Strongly disagree	Disagree	Agree	Strongly agree
Clarity	⬭	⬭	⬭	⬭
Pertinence	⬭	⬭	⬭	⬭
Relevance	⬭	⬭	⬭	⬭

95. Please use the space below to provide your comments, criticisms, or suggestions regarding the evaluated indicator, particularly in terms of semantics, question clarity, wording, and coherence among its components.

96. **Indicator 1:** Establishment of coordination mechanisms and shared care for tuberculosis between social assistance services and services of the Health Care Network*

Measure 2: Shared care for tuberculosis between health and social assistance services

Parameter: Likert Scale:

Satisfactory: 3.6 to 5.0 (always/almost always)

Fair: 2.5 to 3.5 (sometimes)

Unsatisfactory: 1.0 to 2.4 (never/almost never)

Data source: Interviews with health professionals, social assistance professionals, health and/or social assistance managers, and coordinators of the Tuberculosis Control Program (primary data)

Question: *Does shared care for tuberculosis occur between health and social assistance services?*

Please mark only one option per row.

	Strongly disagree	Disagree	Agree	Strongly agree
Clarity	◯	◯	◯	◯
Pertinence	◯	◯	◯	◯
Relevance	◯	◯	◯	◯

97. Please use the space below to provide your comments, criticisms, or suggestions regarding the evaluated indicator, particularly in terms of semantics, question clarity, wording, and coherence among its components.

98. **Indicator 1:** Establishment of coordination mechanisms and shared care for tuberculosis between social assistance services and services of the Health Care Network*

Measure 3: Inclusion of tuberculosis in the work agendas of social assistance

Parameter: Likert Scale:

Satisfactory: 3.6 to 5.0 (always/almost always)

Fair: 2.5 to 3.5 (sometimes)

Unsatisfactory: 1.0 to 2.4 (never/almost never)

Data source: Interviews with health professionals, social assistance professionals, health and/or social assistance managers, and coordinators of the Tuberculosis Control Program (primary data)

Question: *Is tuberculosis included in the work agendas of the municipality's social assistance services?*

Please mark only one option per row.

	Strongly disagree	Disagree	Agree	Strongly agree
Clarity	⬭	⬭	⬭	⬭
Pertinence	⬭	⬭	⬭	⬭
Relevance	⬭	⬭	⬭	⬭

99. Please use the space below to provide your comments, criticisms, or suggestions regarding the evaluated indicator, particularly in terms of semantics, question clarity, wording, and coherence among its components.

100. **Indicator 1:** Establishment of coordination mechanisms and shared care for tuberculosis between social assistance services and services of the Health Care Network*

Measure 4: Inclusion of social protection in the work agendas of the Municipal Tuberculosis Control Program

Parameter: Likert Scale:

Satisfactory: 3.6 to 5.0 (always/almost always)

Fair: 2.5 to 3.5 (sometimes)

Unsatisfactory: 1.0 to 2.4 (never/almost never)

Data source: Interviews with health professionals, social assistance professionals, health and/or social assistance managers, and coordinators of the Tuberculosis Control Program (primary data)

Question: *Is the topic of social protection included in the work agendas of the Municipal Tuberculosis Control Program?*

Please mark only one option per row.

	Strongly disagree	Disagree	Agree	Strongly agree
Clarity	◯	◯	◯	◯
Pertinence	◯	◯	◯	◯
Relevance	◯	◯	◯	◯

101. Please use the space below to provide your comments, criticisms, or suggestions regarding the evaluated indicator, particularly in terms of semantics, question clarity, wording, and coherence among its components.

Subdimension: Intersectoral Actions

102. **Indicator 1:** Management and organization of practices for the implementation, monitoring, and evaluation of intersectoral actions to address tuberculosis, especially among people in situations of social vulnerability*

Measure 1: Coordination between the Municipal Department of Social Assistance and the Municipal Department of Health to address tuberculosis

Parameter: Likert Scale:

Satisfactory: 3.6 to 5.0 (always/almost always)

Fair: 2.5 to 3.5 (sometimes)

Unsatisfactory: 1.0 to 2.4 (never/almost never)

Data source: Interview with health and/or social assistance management professionals and the coordination of the Tuberculosis Control Program (primary data)

Question: *How often does coordination occur between the Municipal Department of Social Assistance and the Municipal Department of Health to address tuberculosis?*

Please mark only one option per row.

	Strongly disagree	Disagree	Agree	Strongly agree
Clarity	◯	◯	◯	◯
Pertinence	◯	◯	◯	◯
Relevance	◯	◯	◯	◯

103. Please use the space below to provide your comments, criticisms, or suggestions regarding the evaluated indicator, particularly in terms of semantics, question clarity, wording, and coherence among its components.

104. **Indicator 1:** Management and organization of practices for the implementation, monitoring, and evaluation of intersectoral actions to address tuberculosis, especially among people in situations of social vulnerability*

Measure 2: Coordination with NGOs, religious institutions, and/or community organizations to address tuberculosis

Parameter: Likert Scale:

Satisfactory: 3.6 to 5.0 (always/almost always)

Fair: 2.5 to 3.5 (sometimes)

Unsatisfactory: 1.0 to 2.4 (never/almost never)

Data source: Interview with health professionals, social assistance professionals, health and/or social assistance managers, and the coordination of the Tuberculosis Control Program (primary data)

Question: *Is coordination with NGOs, religious institutions, and/or community organizations to address tuberculosis a routine practice?*

Please mark only one option per row.

	Strongly disagree	Disagree	Agree	Strongly agree
Clarity	⬭	⬭	⬭	⬭
Pertinence	⬭	⬭	⬭	⬭
Relevance	⬭	⬭	⬭	⬭

105. Please use the space below to provide your comments, criticisms, or suggestions regarding the evaluated indicator, particularly in terms of semantics, question clarity, wording, and coherence among its components.

106. **Indicator 1:** Management and organization of practices for the implementation, monitoring, and evaluation of intersectoral actions to address tuberculosis, especially among people in situations of social vulnerability*

Measure 3: Coordination with strategic areas such as (1) Primary Health Care, (2) Mental Health, (3) Occupational Health, (4) Prison System, (5) Street Outreach Teams for addressing tuberculosis

Parameter: Likert Scale:

Satisfactory: 3.6 to 5.0 (always/almost always)

Fair: 2.5 to 3.5 (sometimes)

Unsatisfactory: 1.0 to 2.4 (never/almost never)

Data source: Interview with health and/or social assistance managers and the coordination of the Tuberculosis Control Program (primary data)

Question: *Are there coordinated efforts with strategic areas such as (1) Primary Health Care, (2) Mental Health, (3) Occupational Health, (4) Prison System, (5) Street Outreach Teams to address tuberculosis?*

Please mark only one option per row.

	Strongly disagree	Disagree	Agree	Strongly agree
Clarity	◯	◯	◯	◯
Pertinence	◯	◯	◯	◯
Relevance	◯	◯	◯	◯

107. Please use the space below to provide your comments, criticisms, or suggestions regarding the evaluated indicator, particularly in terms of semantics, question clarity, wording, and coherence among its components.

Subdimension: Celerity of Justice

108. **Indicator 1:** Guarantee of expedited judicial and administrative proceedings*

Measure 1: Priority in the processing of judicial and administrative cases in which a person affected by tuberculosis is a party or has an interest

Parameter: Yes/No

Satisfactory: ≥ 75% (yes)

Unsatisfactory: ≤ 74% (no)

Data source: Interviews with people affected by tuberculosis (primary data)

Question: *Currently, have you been able to expedite any judicial or administrative proceeding due to being in treatment for tuberculosis?*

For the denominator: *Do you currently have any ongoing judicial or administrative proceeding?*

Calculation method: Number of people with tuberculosis who obtained expedited judicial or administrative proceedings during treatment / Number of people with tuberculosis in follow-up who have an ongoing proceeding

Please mark only one option per row.

	Strongly disagree	Disagree	Agree	Strongly agree
Clarity	◯	◯	◯	◯
Pertinence	◯	◯	◯	◯
Relevance	◯	◯	◯	◯

109. Please use the space below to provide your comments, criticisms, or suggestions regarding the evaluated indicator, particularly in terms of semantics, question clarity, wording, and coherence among its components.

Thank you for participating in this important stage of our study!

We appreciate your participation in this first round of evaluation of the indicators for the validation of the Analysis and Judgment Matrix of our study.

We will analyze your evaluation and, depending on the responses from the other judges for each indicator, we may send this form again with only the revised/adjusted indicators for a new round of evaluation.

Finally, if you know someone who works on the topic of tuberculosis in connection with social protection, and would like to share their contact information to contribute to the evaluation of the indicators in this study, we would be very grateful!

110. I know someone to recommend (Name and/or e-mail):

Google Formulários
